# DNA Microarray Detection of 18 Important Human Blood Protozoan Species

**DOI:** 10.1371/journal.pntd.0005160

**Published:** 2016-12-02

**Authors:** Mu-Xin Chen, Lin Ai, Jun-Hu Chen, Xin-Yu Feng, Shao-Hong Chen, Yu-Chun Cai, Yan Lu, Xiao-Nong Zhou, Jia-Xu Chen, Wei Hu

**Affiliations:** 1 Department of Microbiology and Microbial Engineering, School of Life Sciences, Fudan University, Shanghai, PR China; 2 National Institute of Parasitic Diseases, Chinese Center for Disease Control and Prevention, WHO Collaborating Center for Tropical Diseases, Key Laboratory of Parasite and Vector Biology, National Health and Family Planning Commission, Shanghai, PR China; Makerere University, UGANDA

## Abstract

**Background:**

Accurate detection of blood protozoa from clinical samples is important for diagnosis, treatment and control of related diseases. In this preliminary study, a novel DNA microarray system was assessed for the detection of *Plasmodium*, *Leishmania*, *Trypanosoma*, *Toxoplasma gondii* and *Babesia* in humans, animals, and vectors, in comparison with microscopy and PCR data. Developing a rapid, simple, and convenient detection method for protozoan detection is an urgent need.

**Methodology/Principal Findings:**

The microarray assay simultaneously identified 18 species of common blood protozoa based on the differences in respective target genes. A total of 20 specific primer pairs and 107 microarray probes were selected according to conserved regions which were designed to identify 18 species in 5 blood protozoan genera. The positive detection rate of the microarray assay was 91.78% (402/438). Sensitivity and specificity for blood protozoan detection ranged from 82.4% (95%CI: 65.9% ~ 98.8%) to 100.0% and 95.1% (95%CI: 93.2% ~ 97.0%) to 100.0%, respectively. Positive predictive value (PPV) and negative predictive value (NPV) ranged from 20.0% (95%CI: 2.5% ~ 37.5%) to 100.0% and 96.8% (95%CI: 95.0% ~ 98.6%) to 100.0%, respectively. Youden index varied from 0.82 to 0.98. The detection limit of the DNA microarrays ranged from 200 to 500 copies/reaction, similar to PCR findings. The concordance rate between microarray data and DNA sequencing results was 100%.

**Conclusions/Significance:**

Overall, the newly developed microarray platform provides a convenient, highly accurate, and reliable clinical assay for the determination of blood protozoan species.

## Author Summary

More than 1 billion people are infected with blood protozoan diseases worldwide. The most common blood protozoa in humans, animals, and vectors include *Plasmodium*, *Leishmania*, *Trypanosoma*, *Toxoplasma gondii* and *Babesia*. Due to similar morphology among different blood protozoan species, misdiagnosis always occurs. Most molecular techniques are only carried out in laboratories, with a small number of samples detected simultaneously. Meanwhile, common detection methods may not be convenient for field investigation of large amounts of samples. In order to better manage blood protozoan infection, proper tools are required for the monitoring of these pathogens. Here, a comprehensive and sensitive DNA microarray was developed and tested, which allowed the parallel detection of 18 blood protozoan species.

## Introduction

Blood protozoa are single-cell organisms that often have flagella, cilia or other structures that help them move [1]. They sometimes form parasitic relationships with humans and cause diseases or infections. The most common blood protozoa in humans, animals, and vectors include *Plasmodium*, *Leishmania*, *Trypanosoma*, *Toxoplasma gondii* and *Babesia* [2–6].

Malaria, caused by one of four *Plasmodium* species (*P*. *falciparum*, *P*. *vivax*, *P*. *malariae*, and *P*. *ovale*) and spread through anopheles mosquitoes. It is the deadliest protozoan diseases with nearly 800,000 deaths yearly [2]. Once *Plasmodium* enters into human body, it matures in the liver and blood cells. Symptoms include fever with chills and rigor followed by excessive sweating. If not detected promptly, it can cause cerebral malaria and even death [7]. Hemoflagellates constitute another important group of blood protozoa, whose family includes two genera, *Leishmania* and *Trypanosoma*, both of which require a blood feeding insect vector for transmission, and can infect humans [8, 9]. Leishmaniasis is caused by one of 20 species of protozoa of the *Leishmania* genus. Individuals infected with leishmaniasis often show signs and symptoms such as sores and/or ulcers, swollen glands, weight loss, fever and/or an enlarged spleen [3]. Trypanosomes are distributed in specific areas mainly dictated by vector distribution. *Trypanosoma brucei rhodesiense* and *T*. *b*. *gambiense* cause Human African Trypanosomiasis (HAT), which is transmitted by the tsetse fly. The disease causes fever, headaches, itching, coordination and balance problems, confusion and/or reduced mental abilities. Sometimes, it affects the central nervous system; without treatment, it can be fatal [10, 11]. *Trypanosoma cruzi*, the causative agent of American trypanosomiasis (Chagas’ disease), is characterized by cardiomyopathy, which may present with arrhythmias, conduction defects, cardiomegaly, thromboembolic events, or congestive heart failure [12, 13]. Toxoplasmosis is caused by *Toxoplasma gondii*, which is caused by consumption of contaminated meat [14]. Sometimes, accidental ingestion of cat stool or possibly unwashed vegetables also can infect this disease [14]. Toxoplasmosis is usually asymptomatic and self-limiting but can have serious or fatal effects on fetuses whose mothers contract the disease during pregnancy or in immuno-compromised individuals [15]. For instance, it may be fatal in individuals infected with HIV, due to encephalitis or necrotizing retinochoroiditis [16, 17]. *Babesia* causes a hemolytic disease known as babesiosis, which causes malaria-like symptoms and is often misdiagnosed as malaria. Approximately 100 species of *Babesia* have been identified, but only a few are documented as humans pathogens [18, 19]. *Babesia microti* is the most common strain associated with human infections [20].

Blood protozoan infection is usually diagnosed by microscopic examination of blood smears [1]. Since such detection depends on operator experience, misdiagnosis is common. To overcome this drawback, conventional PCR assays in combination with amplicon sequencing have been used for sensitive and specific detection of several blood protozoan species [21–25]. Molecular techniques have undergone great improvements in recent years. For example, DNA microarrays have been proposed, as high-density microscopic arrangement of immobilized nucleic acid samples on a glass slide; hybridization with fluorescent probes permits evaluation of gene expression at the genome level [26, 27]. The DNA microarray technique has been successfully applied for a range of biological questions, including human cancer[28], metamorphosis of fruit flies [29], and helminth parasites [30]. Recently, DNA microarrays have also been developed and applied for the assessment of major protozoa that cause human and animals’ diseases [31–33].

DNA microarray is an advanced, large-scale, and high output detection technology, which is able to provide a decent platform to genomics and functional genomics research on blood parasites. Currently, gene microarray is mainly applied for functional gene screening [34], exploring the relationships between blood protozoan and their hosts [35], pathogenesis mechanisms [36], drug resistance and targets [37], diagnostic antigens [38], and molecular vaccine screening [39], all of which have yielded excellent results. However, only few of the multitude of microarray technologies are suitable for clinical application and large-scale epidemiological investigations of blood protozoan infections. For example, Li et al. (2005) [40] designed a DNA array for rapid detection and genotyping of pathogenic microbes responsible for epidemic hemorrhagic fever, Tsutsugamushi disease, leptospirosis, malaria, schistosomiasis, cholera, and hemorrhagic colitis. El-Ashker et al. (2015) [41] reported Babesia, Theileria, and Anaplasma species in cattle using PCR assays, gene sequence analysis and a novel DNA microarray. The above studies only included few species of blood protozoa. Thus, the present study aimed to assess the potential diagnostic value of a novel DNA microarray chip in comparison with microscopy and PCR for the diagnosis of common blood protozoan infections.

## Materials and Methods

STARD Checklist and flowchart could be found in S1 and S2 Figs, respectively.

### Ethical Statement

Ethical clearance for the collection and detection of human samples was obtained from the Ethics Committee of the National Institute of Parasitic Diseases (NIPD), Chinese Center for Disease Control and Prevention (China CDC). The objectives, procedures and potential risks were verbally explained to all participants. Signed written informed consent was obtained from all study participants. The study approval notice is found in (S3 Fig). Animals were handled in accordance with good animal practice strictly according to the Animal Ethics Procedures and Guidelines of the People’s Republic of China. The protocol for sampling from animals had been approved by the Animal Welfare& Ethics Committee of the National Institute of Parasitic Diseases, Chinese Center for Disease Control and Prevention in Shanghai (Permit No: IPD-2012-5) (S3 Fig).

### Samples and blood protozoan specimen

Reference blood protozoan samples were either stored in our laboratory or kindly provided by different partner laboratories (S1 Table). A total of 438 samples from humans, animals and vectors (S2 Table) collected from August 2012 to December 2014 were obtained in Myanmar-Yunnan border, Hainan Province, Xinjiang Uygur Autonomous Region, Gansu Province, Guangzhou and Shenzhen in Guangdong Province, and Shanghai (China), as well as 100 blood samples from healthy individuals (S2 Table). *B*. *microti* was from patients in Yunnan and Shanghai, and *B*. *venatorum* from tick in Heilongjiang; *P*. *vivax*, *P*. *falciparum*, *P*. *knowlesi*, *P*. *malariae*, *P*. *ovale* and mixed species *Plasmodium* were from patients in Yuannan; *L*. *donovani* was from human, sand fly and dog in Xingjiang and Sichuan; *L*. *infantum was* from humans, sand fly and dog in Xingjiang, Sichuan and Gansu; *T*. *gondii* was from goat, human, cougar, cat, deer, and toucan in the USA, France, Canada, Brazil, Costa Rica and China (Qinghai, Yunnan and Guangdong). All blood samples were collected according to patient consent as well as medical ethics norms.

Blood smears were prepared for each clinical blood sample. After drying the slides at ambient, the blood smears were quickly fixed in methanol (99%) for 5 min, then stained with 10% Giemsa staining solution (Sigma–Aldrich Chemie GmbH, Taufkirchen, Germany) for 30 min. The slides were examined under an oil immersion lens at a total magnification of 1000 for presence of blood protozoa. After examining more than 50 microscopic fields, blood protozoa were quantified and expressed as a percentage of infected erythrocytes. Blood samples were stored at -80°C until further processing.

In order to detect *Leishmania* infection, smears of marrow and materials from spleen puncture were dried, following fixed in methanol (99%) for 5 min, then stained with 10% Giemsa staining solution (Sigma–Aldrich Chemie GmbH, Taufkirchen, Germany) for 30 min. The slides were examined under an oil immersion lens for observing amastigotes [42]. Meanwhile, the whole blood (suspected infected *Leishmania* from human and canine) with also have been tested by the dipstick (recombinant k39 antigen-based immunochromatographic strip) to detect anti-Leishmania antibody (the Kalazar Detect, batch JL1019; InBios, Seattle, WA) [43].

Tick and sandfly samples were preserved in 75% ethanol for subsequent DNA extracting and PCR amplification.

### DNA extraction from samples

DNA was extracted from whole blood of human and animals using QIAmp DNA Mini kit 250 (QIAGEN) according to the manufacturer’s instructions. Genomic DNA from marrow, materials from spleen puncture and the vectors (ticks and sandflies) were also extracted by the above kit, which can be used for DNA extracting from both tissue and blood specimens. Positive control samples were used as described in S1 Table. The concentration of DNA was measured on a NanoDrop ND-1000 Spectrophotometer (peQLab Biotechnologie GmbH, Erlangen, Germany).

### Establishment of multiple PCR microarray

#### Specific primers and microarray probe design

18S small subunit ribosomal DNA (18S rDNA), mitochondrial cytochrome c oxidase subunit 1 (pcox1), Internal Transcribed Spacer (ITS) gene sequences, fumarate hydratase (fh) gene, lysosomal/endosomal membrane protein p67 gene and hypothetical protein gene (hp), etc. of 18 blood protozoan species were obtained from GenBank. In Babesia species (*B*. *microti*, *B*. *divergens*, *B*. *duncani*, and *B*. *venatorum*), the 18S rDNA conserved region was selected. For *Plasmodium*, 18S rDNA+ITS were used to amplify *P*. *vivax* and *P*. *falciparum*; pcox1 was used for *P*. *knowlesi*, *P*. *malariae*, and *P*. *ovale*. In *Leishmania* spp., 18S rDNA was selected for *L*. *donovani*, *L*. *gerbilli* / *L*. *tropica*, *L*. *aethiopica* and *L*. *infantum*; fh was for *L*. *gerbilli*; mspC was used for *L*. *tropica*. 18S rDNA was also used to identify *T*. *cruzi* and *T*. *brucei*. Moreover, *T*. *b*. *rhodesiense* and *T*. *b*. *gambiense* were distinguished by p67 and hp gene, respectively. *T*. *gondii* was amplified by primers designed according to the conserved region of pcox1. Then, Primer 5.0 and Array Designer 4.2 was used to design specific primers and probes for each gene fragment (S3 and S4 Tables).

The microarray target probes were selected according to ⊿G (hybridization thermodynamics) which was calculated by the nearest-neighbor method [44] and BLAST sequence analysis. For a probe to be considered as “good” candidate for microarrays, ⊿G must be at least -70 kcal/mol for homologous sequences and more than -40 kcal/mol for heterologous ones. A minimum of 6 non-overlapping probes designed from conserved regions were selected for each genus, and each species of blood protozoa was recognized by at least two probes. Due to variability within species, if necessary, two or more source sequences were selected and processed as described above.

#### Specific PCR amplification

The specific primers in S3 Table were used for PCR amplification, in a total volume of 25 μL containing 2.5 μL of 10×buffer (Mg^2+^ free), 2 μL mixed dNTPs (2.5 mM each) (Takara), 10 pmol of each primer (Sangon Biotech Corporation, Shanghai, China) one unit Taq DNA polymerase (Takara), and 3μL of extracted DNA. PCR assays were performed on a thermocycler (Biometra) at 94°C for 5 min (pre-denaturation), followed by 35 cycles of 94°C for 30 s (denaturation), 55°C for 30 s (annealing), and 72°C for 1 min 30 s (extension), and a final extension of 72°C for 10 min. Samples without genomic DNA (distilled water) were included in each PCR run as ‘negative’ controls. An aliquot (5 μL) of each PCR product was examined by 1% agarose gel electrophoresis; gels were stained with ethidium bromide and photographed on a gel documentation system (UVItec). One amplicon representing each of the species was sequenced to confirm their identity.

#### Multiple PCR DNA microarray preparation and detection

20 pairs of specific primers (S3 Table) were used in DNA fragment amplification of reference strains of *P*. *falciparum*, *P*. *vivax*, *P*. *ovale*, *P*. *malariae*, *P*. *knowlesi*, *L*. *donovani*, *L*. *infantum*, *L*. *tropica*, *L*. *aethiopica*, *L*. *gerbilli*, *T*. *cruzi*, *T*. *b*. *rhodesiense*, *T*. *b*. *gambiense*, *T*. *gondii*, *B*. *microti*, *B*. *divergens*, *B*. *venatorum*, and *B*. *duncani*. Positive amplicons were sequenced directly on an ABI 377 automated DNA sequencer (using Big Dye Terminator Chemistry) employing the above PCR primers (individually).

### DNA detection microarray preparation

**Fig 1:**
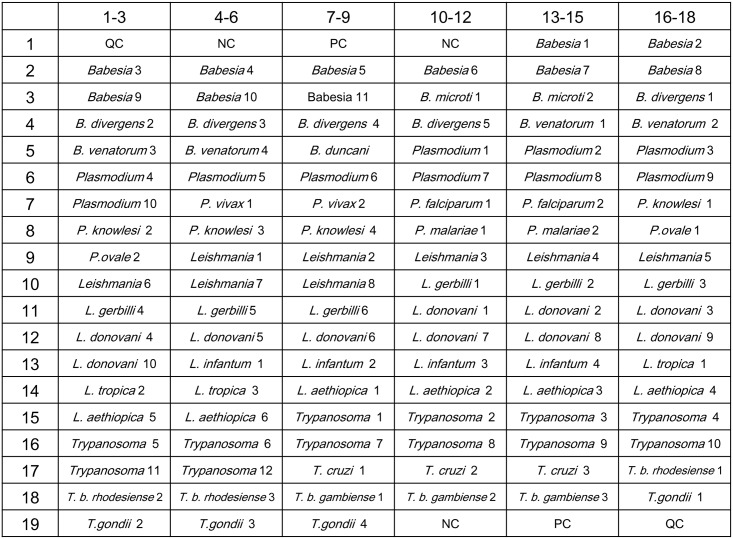
Probe arrangement of the DNA microarray system. *Babesia* 1–11: *Babesia* genus probes; *B*. *microti* 1 & 2: *B*. *microti* species probes; *B*. *divergens* 1–5: *B*. *divergens* species probes; *B*. *venatorum* 1–4: *B*. *venatorum* species probes; *B*. *duncani*: *B*. *duncani* species probes; *Plasmodium* 1–10: *Plasmodium* genus probes; *P*. *vivax* 1 & 2: *P*. *vivax* species probes; *P*. *falciparum* 1 & 2: *P*. *falciparum* species probes; *P*. *knowlesi* 1–4: *P*. *knowlesi* species probes; *P*. *malariae* 1 & 2: *P*. *malariae* species probes; *P*. *ovale* 1 & 2: *P*. *ovale* species probes; *Leishmania* 1–8: *Leishmania* genus probes; *L*. *gerbilli* 1–6: *L*. *gerbilli* species probes; *L*. *donovani* 1–10: *L*. *donovani* species probes; *L*. *infantum* 1–4: *L*. *infantum* species probes; *L*. *tropica* 1–3: *L*. *tropica* species probes; *L*. *aethiopica* 1–6: *L*. *aethiopica* species probes; *Trypanosoma* 1–12: *Trypanosoma* genus probes; *T*. *cruzi* 1–3: *T*. *cruzi* species probes; *T*. *b*. *rhodesiense* 1–3: *T*. *b*. *rhodesiense* species probes; *T*. *b*. *gambiense* 1–3: *T*. *b*. *gambiense* species probes; *T*. *gondii* 1–4: *T*. *gondii* species probes. QC: quality control; PC: positive control; NC: negative control.

Oligonucleotide probes were designed in accordance with multiple-sequence alignment analysis of sequences available in GenBank with the Array Designer 4.2 program. Probes were selected in several species-specific sequence regions of the 18S rDNA, cox1, ITS genes, for differentiation among species. An artificial mismatch was introduced into specific oligonucleotide probes to distinguish the similar species and provide better thermal differentiation between the sequences. The probes were between 40 and 58 nucleotides, with melting temperatures (*T*m) of 60–65°C. The 5^’^ end of each probe was modified by addition of a spacer with 25 consecutive thymine residues and an amino-linker group for covalent immobilization on the aldehyde-coated glass surface. Oligonucleotide probes were contact printed onto OPAldehyde Slide aldehyde-activated slides at a concentration of 10μM in DNA spotting solution on a Smart Arrayer-96 Microarrayer (both from CapitalBio, Beijing, China), and covalently immobilized on the slides via an amino group at their 5’ ends [45–47] to create biochips (Fig 1). All oligonucleotide primers and probes listed in S3 and S4 Tables were obtained from CapitalBio Corporation, Beijing, China. In each array, three types of controls (a surface chemistry control: fluorescent dye HEX-labeled oligonucleotide, a hybridization positive control to monitor the hybridization process: oligonucleotide complementary to a synthetic template, and the negative control for background signal corrections: oligonucleotide designed to not hybridize to any sequence present) were printed.

### Microarray probe in silico analysis

The hybridization thermodynamics of probes have been evaluated *in silico* with reference blood protozoan isolates shown in Table 1. The hybridization ⊿G (kcal/mol) between probe and target was calculated by the nearest-neighbor method. Detection of a target was achieved at⊿G < -50 kcal/mol [48].

**Table 1:** Reference blood protozoan species used in microarray validation.

Family	Genus	Species	Strain	No. of positive genus probes/ total genus	No. of positive species probes/ total species
Babesiidae	*Babesia*	*Babesia microti*	ATCC PRA-99TM	3/11	2/2
		*Babesia divergens*	Clinical reference	1/11	5/5
		*Babesia venatorum*	Clinical reference	3/11	4/4
		*Babesia duncani*	Clinical reference	4/11	1/1
Plasmodidae	*Plasmodium*	*Plasmodium vivax*	Clinical reference	2/10	2/2
		*Plasmodium falciparum*	3D7	4/10	2/2
		*Plasmodium knowlesi*	Clinical reference	1/10	4/4
		*Plasmodium malariae*	Clinical reference	2/10	2/2
		*Plasmodium ovale*	Clinical reference	2/10	2/2
Trypanosomatidae	*Leishmania*	*Leishmania gerbilli*	MRHO/CN/60/GERBILLI	2/8	6/6
		*Leishmania donovani*	MHOM/IN/80/DD8	5/8	10/10
		*Leishmania infantum*	MHOM/CN/86/SC6	1/8	4/4
		*Leishmania tropica*	MHOM/SU/74/K27	2/8	3/3
		*Leishmania aethiopica*	MHOM/ET/72/L100	2/8	6/6
Trypanosomatidae	*Trypanosoma*	*Trypanosoma cruzi*	Clinical reference	7/12	2/3
		*Trypanosoma brucei rhodesiense*	YTAT 1.1 PF	3/12	3/3
		*Trypanosoma brucei gambiense*	Clinical reference	3/12	3/3
Sarcocystidae	*Toxoplasma*	*Toxoplasma gondii*	TgCatBr5	\	4/4

### Microarray production

Illumina Oligator (Illumina Inc., CA, USA) was used to synthesize probes. Then, oligonucleotides were resuspended to 400 pmol in 3×SSC buffer (0.45 M NaCl, 45 mM sodium citrate, pH 7.0), following spotted onto epoxide-coated glass slides in the Microarray Facility of CapitalBio Corporation. One specific probe was contained in each spot that can detect one blood protozoan species. Slides were stored in a humidity-free chamber until use.

Coupling reactions of sample DNA with Cy3 as well as probes with Cy5 dyes (GE HealthCare, USA) were carried out as described elsewhere [49]. Fluorophore-labeled DNA was purified with a Zymo DNA Clean & Concentrator-5 kit, and label incorporation was quantified on a NanoDrop system.

### Slide preparation, hybridization and scanning

Before use, the microarray slides were washed with an ethanolamine washing solution (50 mM ethanolamine, 0.1% SDS, 0.1 M Tris, pH 9) for 15 min at 50°C, then washed twice with distilled water, and dried by centrifugation for 5 min at 500 rpm. The processed slides were loaded with 30 μL of a combination of Cy3- and Cy5-labeled DNA in 3×SSC buffer, and hybridization was allowed in a sealed chamber which submerged in a water bath at 65°C for 8-12 h. Next step, the slides were washed consecutively in 2×SSC (65°C), 2×SSC, 1×SSC, and 0.2×SSC after incubation, and dried for 5 min at 500 rpm. Hybridization images were acquired with an AxonGenePix 4000B scanner (Molecular Devices, USA) and synchronized with the GenePix Pro 6.0 software for spot intensity assessment.

### Data analysis

The intensity of each hybridization spot was first filtered by spot size and shape (denoted as good/bad/absent), foreground signal saturation percentage on channel 532 (denoted as F532, <5 was considered to be satisfactory), background signal saturation percentage on channel 532 (denoted as B532), and the F532 to B532 ratio [(% >B532 + 2 standard deviations), >50%] to determine hybridization quality. Good quality spots were used to generate microarray level background values. Normalization of intensity values was performed according to the formula (F532i/F532m)—(B532i-B532m), where i represents each individual spot, m is the sum of all spots. Thus, F532i and B532i represented as foreground and background signals of a spot “i,” respectively, while F532m and B532m are the sums of all foreground or background spots, respectively.

In reference samples, statistical significance of probe intensities was assessed by the rank products algorithm [50], using a minimum of three technical replicates. A “spot rank value” from negative-controls was used in values analyzed with the versatile R package [51] which can estimate local and tail area-based false discovery rates (FDR) [52]. Positive blood protozoan species were defined as having at least two probes with P<0.05 and FDR<0.01.

### Limit-of-detection assays

The purified corresponding gene fragments (S3 Table) of *Plasmodium*, *Leishmania*, *Trypanosoma*, *T*. *gondii*, and *Babesia*, among others, were ligated into the pMD-18 T vector, and transformed into *Escherichia coli* JM109. Positive clones were screened, and the positive plasmid extracted. The amounts of gene-positive plasmids were detected on a nucleic acid micro-analyzer (CapitalBio Corporation, Beijing, China); copy number was calculated according to the following formula: (6.02×10^23^) × (ng/μL×10^−9^)/ (length of DNA fragment×660) = copy/μL [53]. Dilutions of positive plasmids corresponding to 1×10^6^ to 10 particles were analyzed alone and amplified, labeled, and processed using the DNA microarray protocol described above.

## Results

An advantage of the microarray technology is its capacity to assess hundreds and even thousands of targets in a single detection. The main goal of this research was to develop a chip for the detection of 18 species of blood protozoa including *Plasmodium*, *Leishmania*, *Trypanosoma*, *T*. *gondii*, and *Babesia* found in humans, animals, and vectors, which should be convenient used in clinical and epidemiological studies in humans and animals.

### Screening and verification of specific primers and probes

A set of 20 pairs of specific primers (S3 Table) and 107 microarray probes (S4 Table) were selected and designed to identify 18 species in 5 genera of blood protozoa associated with humans, animals and vectors. The electrophoretograms of representative PCR products and multiple PCR products for the 18 species of blood protozoa are shown in S4 and S5 Figs. The highest number of probes covered *Plasmodium* (22 probes), *Leishmania* (37 probes), *Trypanosoma* (21probes), *T*. *gondii* (4 probes), and *Babesia* (23 probes) (S4 Table).

**Fig 2:**
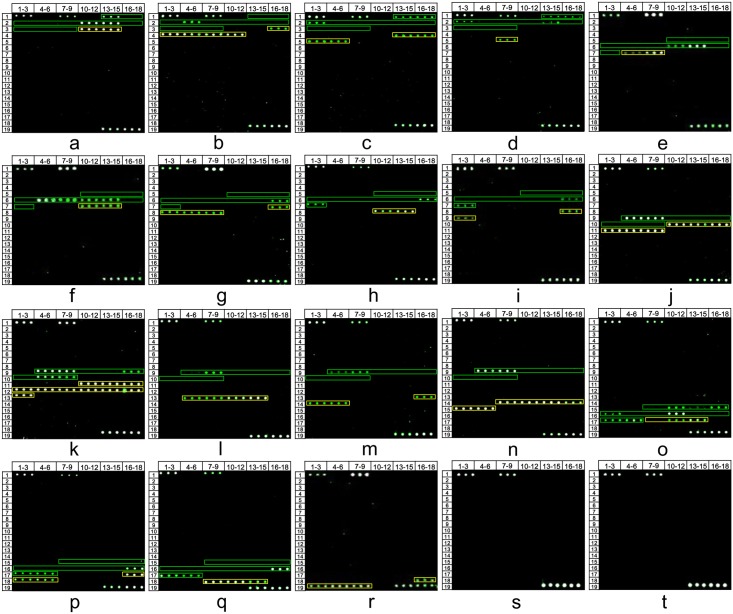
Blood protozoan detection DNA microarray results for 18 blood protozoan species and samples from healthy adults. a-t: Blood protozoan detection DNA microarray data for *B*. *microti*, *B*. *divergens*, *B*. *venatorum*, *B*. *duncani*, *P*. *vivax*, *P*. *falciparu*m, *P*. *knowlesi*, *P*. *malariae*, *P*. *ovale*, *L*. *gerbilli*, *L*. *donovani*, *L*. *infantum*, *L*. *tropica*, *L*. *aethiopica*, *T*. *cruzi*, *T*. *b*. *rhodesiense*, *T*. *b*. *gambiense*, and *T*. *gondii*, healthy adult and negative control.

Reference strains for 18 species of blood protozoa were available for probe validation. These species represent 5 genera, and included 5 human *Plasmodium*, 4 human *Leishmania*, 2 human *Trypanosoma*, 1 human *Babesia*, 1 sand rat *Leishmania*, 1 rat *Trypanosoma*, 1 cat *T*. *gondii*, 1 field mouse *Babesia* and 2 tick *Babesia* (S1 Table and Table 1). All reference strains were detected as expected; the number of positive species and probes in each genus are shown in Table 1. Microarray showed no hybridization fluorescence signal, indicating a good specificity for this microarray assay (Fig 2).

### Assay sensitivity and specificity

**Fig 3:**
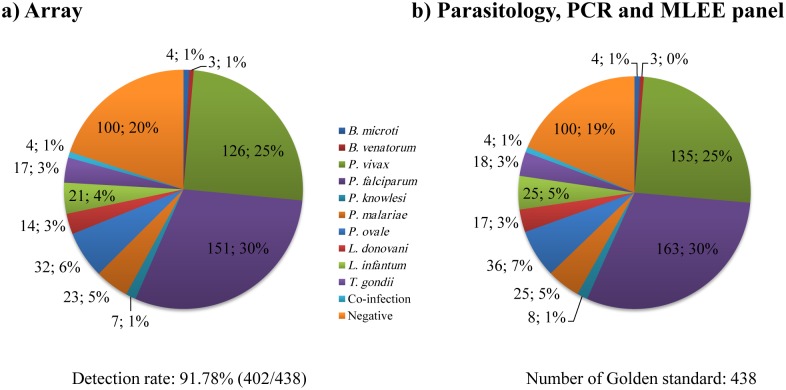
Microarray detection of blood protozoa isolates or clinical samples from infected blood protozoan diseases. a. Microarray detection for 438 blood protozoan samples from infected blood from cases with protozoa and 100 healthy adults; b. A parallel detection by morphology, PCR sequencing and multi-locus enzyme electrophoresis was achieved.

**Fig 4:**
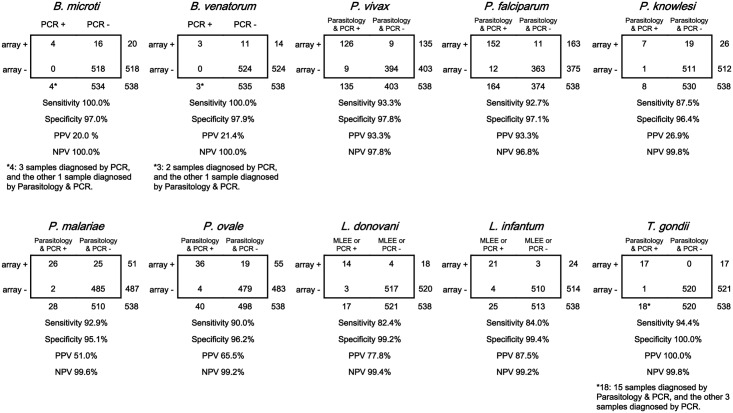
Sensitivity and specificity of DNA microarray for detecting blood protozoa.

To further assess the value of the developed microarray assay in detecting blood protozoa, 438 blood samples (including 4 samples from cases with combined infection, i.e. 3 combined *P*. *malariae and P*. *ovale*, and one case with combined infection of *P*. *falciparum and P*. *ovale*), collected from 2012 to 2014 in China, were analyzed (S2 Table) alongside and 100 samples from healthy adults. Blood protozoan reference isolates were detected by DNA microarray, while performing parallel evaluation using gold standard methods such as morphology, PCR or multi-locus enzyme electrophoresis (MLEE). The results are shown in Fig 3. Compared to the “gold standard methods”, the positive detection rate of the microarray assay was 91.78% (402/438). Detection rates were 100.0% (3/3), 100.0% (4/4), 93.3% (126/135), and 92.7% (152/164, including 151 cases only infected with *P*. *falciparum*; one with the combined infection of *P*. *falciparum and P*. *ovale*), 87.5% (7/8), 92.9% (26/28, including 23 cases only infected with *P*. *malariae*; 3 cases with the combined infection of *P*. *malariae and P*. *ovale*), 90.0% (36/40, including 32 cases only infected with *P*. *ovale*; 4 with combined infection of *P*. *ovale and P*. *malariae* or *P*. *falciparum*), 82.4% (14/17), 84.0% (21/25), and 94.4% (17/18) in the samples for *B*. *microti*, *B*. *venatorum*, *P*. *vivax*, *P*. *falciparum*, *P*. *knowlesi*, *P*. *malariae*, *P*. *ovale*, *L*. *donovani*, *L*. *infantum*, and *T*. *gondii*, respectively (Figs 3, 4 and Table 2).

**Table 2:** Sensitivity, specificity, positive predictive value (PPV), negative predictive value (NPV) and Youden index of blood protozoan detection DNA microarrays.

	*B*. *microti*	*B*. *divergens*	*B*. *venatorum*	*B*. *duncani*	*P*. *vivax*	*P*. *falciparum*	*P*. *knowlesi*	*P*. *malariae*	*P*. *ovale*	*L*. *gerbilli*	*L*. *donovani*	*L*. *infantum*	*L*. *tropica*	*L*. *aethiopica*	*T*. *cruzi*	*T*. *b*. *rhodesiense*	*T*. *b*. *gambiense*	*T*.*gondii*
No. of true positives	4	0	3	0	126	152	7	26	36	0	14	21	0	0	0	0	0	17
No. of false positives	16	11	11	12	9	11	19	25	19	7	4	3	5	4	3	2	3	0
No. of true negatives	518	527	524	526	394	363	511	485	479	531	517	510	533	534	535	536	535	520
No. of false negatives	0	0	0	0	9	12	1	2	4	0	3	4	0	0	0	0	0	1
Sensitivity (%) [95% CI*]	100.0	\**	100.0	\	93.3 [89.1–97.5]	92.7 [88.8–96.5]	87.5 [66.1–100.0]	92.9 [83.7–100.0]	90.0 [81.2–98.8]	\	82.4 [65.9–98.8]	84.0 [70.8–97.2]	\	\	\	\	\	94.4 [84.2–100.0]
Specificity (%) [95% CI]	97.0 [95.6–98.4]	98.0 [96.8–99.2]	97.9 [96.7–99.1]	97.8 [96.5–99.0]	97.8 [96.3–99.2]	97.1 [95.3–98.8]	96.4 [94.8–98.0]	95.1 [93.2–97.0]	96.2 [94.5–97.9]	98.7 [97.7–99.7]	99.2 [98.5–100.0]	99.4 [98.8–100.0]	99.1 [98.3–99.9]	99.3 [98.5–100.0]	99.4 [98.8–100.0]	99.6 [99.1–100.0]	99.4 [98.8–100.0]	100.0
PPV (%) [95% CI]	20.0 [2.5–37.5]	\	21.4 [0–42.9]	\	93.3 [89.1–97.5]	93.3 [89.4–97.1]	26.9 [9.9–44.0]	51.0 [37.3–64.7]	65.5 [52.9–78.0]	\	77.8 [58.6–97.0]	87.5 [74.3–100.0]	\	\	\	\	\	100.0
NPV (%) [95% CI]	100.0	100.0	100.0	100.0	97.8 [96.3–99.2]	96.8 [95.0–98.6]	99.8 [99.4–100.0]	99.6 [99.0–100.0]	99.2 [98.4–100.0]	100.0	99.4 [98.8–100.0]	99.2 [98.5–100.0]	100.0	100.0	100.0	100.0	100.0	99.8 [99.4–100.0]
Youden’s index	0.97	\	0.98	\	0.91	0.90	0.84	0.88	0.86	\	0.82	0.83	\	\	\	\	\	0.94

* CI: confidence interval

** \: No analysis

**Fig 5:**
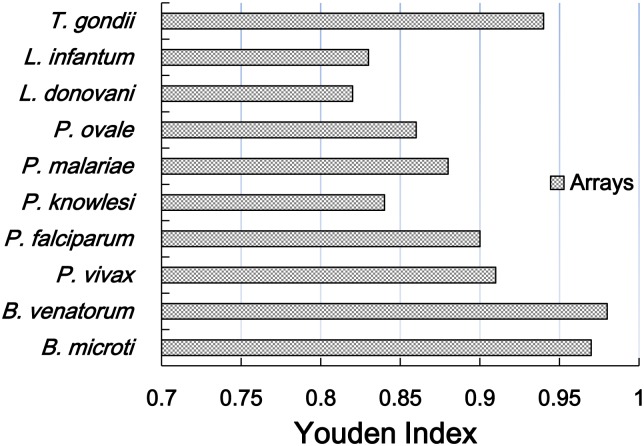
Youden index of DNA microarray for the detection of blood protozoa.

Sensitivity and specificity of the blood protozoa detection were 82.4% (95%CI 65.9% ~ 98.8%) to 100.0% and 95.1%CI (95% CI 93.2%~97.0%) to 100.0%, respectively. Positive predictive value (PPV) and negative predictive value (NPV) were 20.0% (95% CI 2.5% ~ 37.5%) to 100.0% and 96.8% (95% CI: 95.0% ~ 98.6%) to 100.0%, respectively. Youden index values were from 0.82 to 0.97 (Figs 4, 5 and Table 2).

### Detection limit

To determine the detection limit of the PCR and DNA microarray assay, the concentrations of specific gene-positive plasmids of *Plasmodium*, *Leishmania*, *Trypanosoma*, *T*. *gondii*, and *Babesia* were assessed. The detection limit of the DNA microarray was between 200 to 500 copies/reaction, similar to PCR data (Table 3).

**Table 3:** Detection limits of DNA microarray and PCR.

Species	DNA microarray(copies/reaction)	PCR(copies/ reaction)
*Babesia microti*	200	200
*Babesia divergens*	500	500
*Babesia venatorum*	500	500
*Babesia duncani*	500	500
*Plasmodium vivax*	200	200
*Plasmodium falciparum*	200	200
*Plasmodium knowlesi*	500	500
*Plasmodium malariae*	500	500
*Plasmodium ovale*	200	200
*Leishmania gerbilli*	200	200
*Leishmania donovani*	500	500
*Leishmania infantum*	500	500
*Leishmania tropica*	500	500
*Leishmania aethiopica*	200	200
*Trypanosoma cruzi*	500	500
*Trypanosoma brucei rhodesiense*	500	500
*Trypanosoma brucei gambiense*	500	500
*Toxoplasma gondii*	200	200

### Repeatability of the DNA microarray assay

A total of 5 microarrays from each batch (20150925, 20151026, 20151125, 20151224, and 20160125) were selected randomly for the detection of reference blood protozoa in the same conditions as described above. Detection results of the reference isolates were positive (S6 and S7 Figs), indicating that the DNA microarray assay developed had good repeatability.

## Discussion

The current routine blood protozoa testing method is morphology. Due to similar morphology among different species of blood protozoa (for example *Plasmodium* and *Babesia*) and the technical variations for detection operators, misdiagnosis is common [1]. Recently, the number of blood protozoa identified has increased considerably for applying the genomic technologies, e.g. next-generation sequencing technologies with the principle of Sequencing by Synthesis, including the platform of Roche/454FLX, Illumina/Solexa Genome Analyzer, and Applied Biosystems SOLID system in studies of genetic, fatal or rare cases of diseases affecting humans and animals [41, 54]. Most molecular technologies are only carried out in laboratories, with only a few samples detected simultaneously. Therefore, common detection methods may not be convenient in field investigation for large amounts of samples [1]. To better assess infections by blood protozoa, proper detection methods are required for pathogenic monitoring. Here, a sensitive and comprehensive DNA microarray assay was developed with the purpose of the parallel detection of 18 blood protozoan species.

A PCR microarray is a solid carrier with several primers of known genes, and used to assess gene expression by the PCR technique. The PCR microarray technology is a high-throughput method with accuracy and sensitivity, and different from the microarray technology based on hybridization. The advantages of PCR microarray over conventional PCR are: (a). Integrity. The operating system of PCR microarrays applies multi-gene amplification with the integration of result analysis and easy operation. However, traditional PCR employs single gene amplification and data analysis, and its operation is complicated. (b). Time. Time of PCR microarray detection is greatly reduced. (c). Regents. Higher amounts of reagents are required in conventional PCR, and it is difficult to obtain the desired amplification under 15 μl reaction volume. Meanwhile, PCR microarrays can amplify 10 μL sample or less. This could reduce the amounts of reagents, e.g. Taq polymerase, saving experimental cost and reducing waste pollution[55, 56]. Overall, the PCR microarray technology has the advantages of small size, fast response, simple operation, low cost, sample saving, no pollution, and easy integration[57, 58].

Design and implementation of a DNA microarrayto detect and identify blood protozoa is not an easy task. Probe design and experimental conditions are two important parameters in careful consideration. Resequencing microarrays, which permit the identification of mutations, require numerous probes for a single gene, increasing the overall cost [59]. In this research, gene sequences of 18 blood protozoan species in the GenBank were analyzed. A total of 20 pairs of specific primers and 107 probes of blood protozoa were successfully designed according to the specific gene fragments. Conventional PCR verification was carried out by amplifying the target genes of reference blood protozoan isolates, with results similar to microarray findings.

Some DNA microarrays have been previously reported in the identification of main blood protozoa [31–33]. However, the latter targeted mostly the identification of one or two species. The microarray method described in this work was validated with 18 species of reference blood protozoa. Importantly, 438 samples corresponding to the infected areas were detected. The microarray assay can readily accommodate numerous probes and could easily increase the information to resolve more protozoa species. Considering the prevalence of blood protozoan species found in Chinese patients, the present DNA microarray technique could easily meet the requirements for clinical detection. However, with further development, additional probes could be included to detect more species to further the scope of CDC epidemiological investiagtions.

Moreover, the microarray system presented in this research has many major advantages that could be used in clinical practice greatly, particularly the ample quality controls (three control types as discussed in Materials and Methods), global signal uniformity which can emerges higher quality hybridization results, and semi-automation and total solution-based procedures, which also meet the requirement of high-throughput from busy clinics.

One of the important parameters in blood protozoan detection is assay sensitivity, which can be affected by several factors. In the case of microarrays, nucleic acids in samples are processed by random-primed amplification before hybridization to ensure amplification of various blood protozoa [60]. The specificyt of random PCR is lower than that of specific PCR, decreasing assay sensitivity, as all as genetic materials are amplified, and diluted the positive signals [60]. However, the limit of detection of the current blood protozoan detection method was 200 to 500 copies/reaction, indicating a good sensitivity.

In this research, a group of clinical samples collected from patients with fever, as well as animals and vectors were subsequently analyzed. Firstly, the clinical samples were tested by morphology, which led to the identification of *Plasmodium* and *Babesia*; the microarray technique could distinguish them. it suggested that the latter platform owns a higher specificity than traditional methods.

Parallel detection of blood protozoan species should increase understanding of their etiologies, as it can increase the detection rate of positive cases. Furthermore, it may close the diagnostic gap that represents an important factor. Additional data from case-control studies and parameters from other hosts, such as serological data, would help provide evidence for blood protozoan pathogenicity. Moreover, more epidemiological studies in animals and vectors should be considered.

In addition to high specificity, accuracy, sensitivity, and increased information, we believe that the present microarray system will have several other critical advantages. Automatic chip washing procedures as well as biochip scanner and data software were introduced with the aim to reduce operators’ manual work burden and ease implementation of the technique into routine workflow. The amplified target DNA fragments were detected in the newly developed assay after fluorescently labeled. Furthermore, there was only a single washing step needed after hybridization by automatic chip cleanup instrument. At last, For the data acquisition from biochip was analyzed by a dedicated software, with results printed automatically.

Although the newly designed microarray platform has a number of overt advantages, It was also recognized that there is room for further improvement and development. We will pay attention to the more discriminatory loci assessed to ensure that individual species are unequivocally identified. Further development of the workstation is required to fully automatize the procedure; this would also have direct benefits to workflows, particularly in larger test institutes. In conclusion, the microarray platform can provide a convenient, accurate and reliable diagnostic tool for the identification of 18 most common blood protozoan species. The system should be widely used in future detection assay and management of blood protozoan infections in busy hospitals and research institutes, and would help in disease control and prevention plans.

## Supporting Information

S1 Fig STARD Checklist. (PDF)

S2 Fig Flowchart. (TIF)

S3 Fig Ethical clearance for the collection and examination of human and animal serum. (PDF)

S4 Fig Representative PCR products for 18 blood protozoan species. a-v: Representative PCR products for *B*. *microti*, *B*. *divergens*, *B*. *duncani*, *B*. *venatorum*, *P*. *vivax*, *P*. *falciparu*m, *P*. *knowlesi*, *P*. *malariae*, *P*. *ovale*, *L*. *donovani*, *L*. *gerbilli* (18S rDNA gene), *L*. *gerbilli* (fumarate hydratase gene), *L*. *tropica* (18S rDNA gene), *L*. *tropica* (mspC gene), *L*. *infantum*, *L*. *aethiopica*, *T*. *b*. *rhodesiense* (18S rDNA gene), *T*. *b*. *rhodesiense* (lysosomal/endosomal membrane protein p67 gene), *T*. *b*. *gambiense* (18S rDNA gene), *T*. *b*. *gambiense* (hypothetical protein gene), *T*. *cruzi* and *T*. *gondii*. M: Marker; 1: positive control; 2: negative; 3: blood of health human; 4: reference sample.
(TIF)

S5 Fig Representative multiple PCR products for 18 blood protozoan species. M: Marker; 1: positive control; 2: negative; 3: *B*. *microti*; 4: *B*. *divergens*; 5: *B*. *duncani*; 6: *B*. *venatorum*; 7: *P*. *vivax*; 8: *P*. *falciparu*m; 9: *P*. *knowlesi*; 10: *P*. *malariae*; 11: *P*. *ovale*; 12: *L*. *donovani*; 13: *L*. *gerbilli*; 14: *L*. *tropica*; 15: *L*. *infantum*; 16: *L*. *aethiopica*; 17: *T*. *b*. *rhodesiense*; 18: *T*. *b*. *gambiense*; 19: *T*. *cruzi*; 20: *T*. *gondii*; 21: healthy adult.
(TIF)

S6 Fig Repeatability of DNA microarray of *Babesia microti*. 1–5: 5 times repeatability of DNA microarray of *B. microti* with the array of 20150925 batche; 6–10: 5 times repeatability of DNA microarray of *B. microti* with the array of 20151026 batche; 11–15: 5 times repeatability of DNA microarray of *B. microti* with the array of 20151125 batche; 16–20: 5 times repeatability of DNA microarray of *B. microti* with the array of 20151224 batche; 21–25: 5 times repeatability of DNA microarray of *B. microti* with the array of 20160125 batche.
(TIF)

S7 Fig Repeatability of DNA microarray of *Plasmodium falciparum*. 1–5: 5 times repeatability of DNA microarray of *P. falciparum* with the array of 20150925 batche; 6–10: 5 times repeatability of DNA microarray of *P. falciparum* with the array of 20151026 batche; 11–15: 5 times repeatability of DNA microarray of *P. falciparum* with the array of 20151125 batche; 16–20: 5 times repeatability of DNA microarray of *P. falciparum* with the array of 20151224 batche; 21–25: 5 times repeatability of DNA microarray of *P. falciparum* with the array of 20160125 batche.
(TIF)

S1 Table Reference blood protozoan samples. (DOCX)

S2 Table Clinical protozoan isolates from reference blood. (DOCX)

S3 Table Specific primers for blood protozoan amplification. (DOCX)

S4 Table Probes for blood protozoan detection. (DOCX)
