# Supplementary figures and images for: DNA Microarray Detection of 18 Important Human Blood Protozoan Species
Source: PLoS Negl Trop Dis. 2016 Dec 2;10(12):e0005160. doi: 10.1371/journal.pntd.0005160 (PMC5135439; doi:10.1371/journal.pntd.0005160)

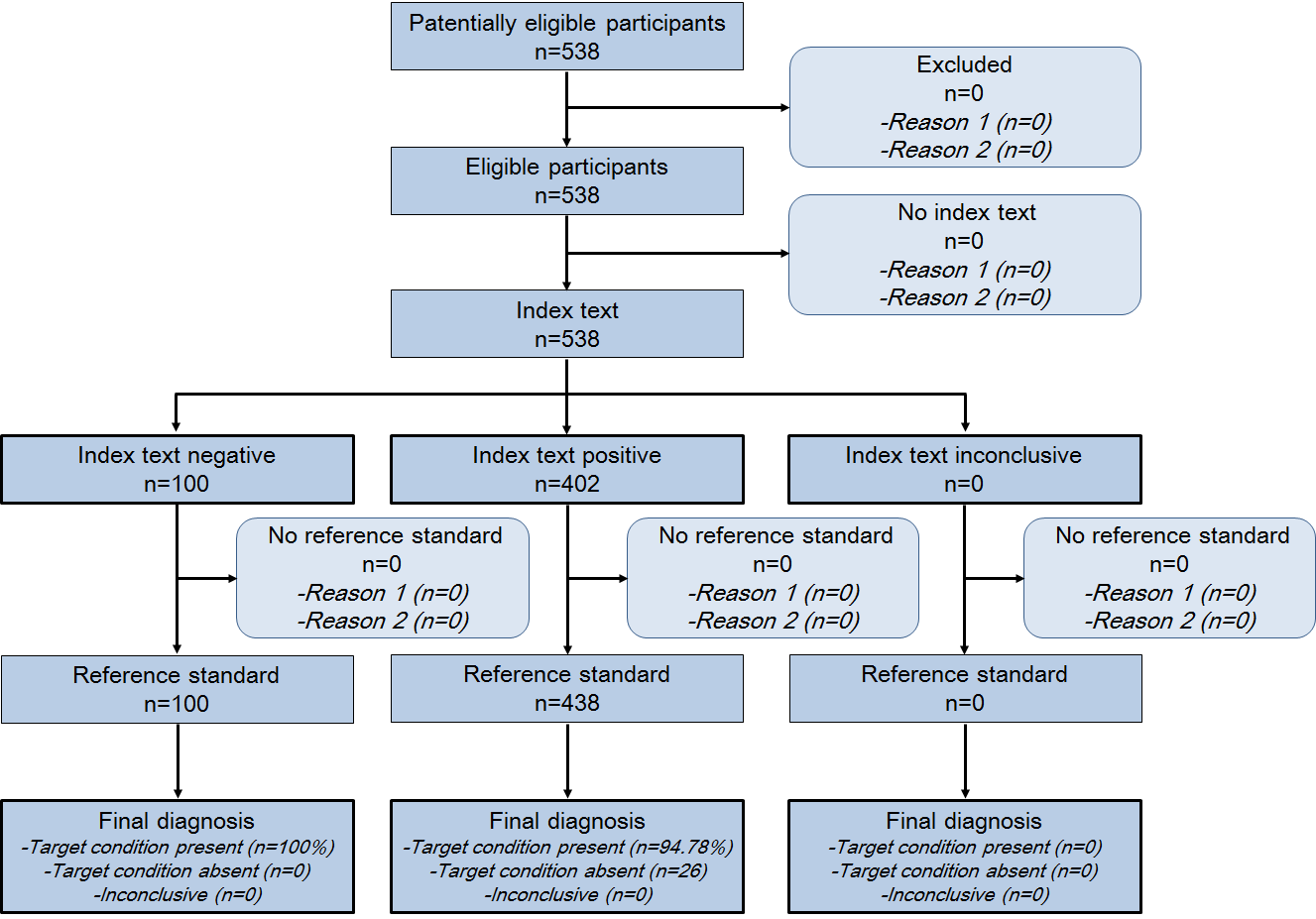

Supplement: S2 Fig — (TIF) [file pntd.0005160.s002.tif]

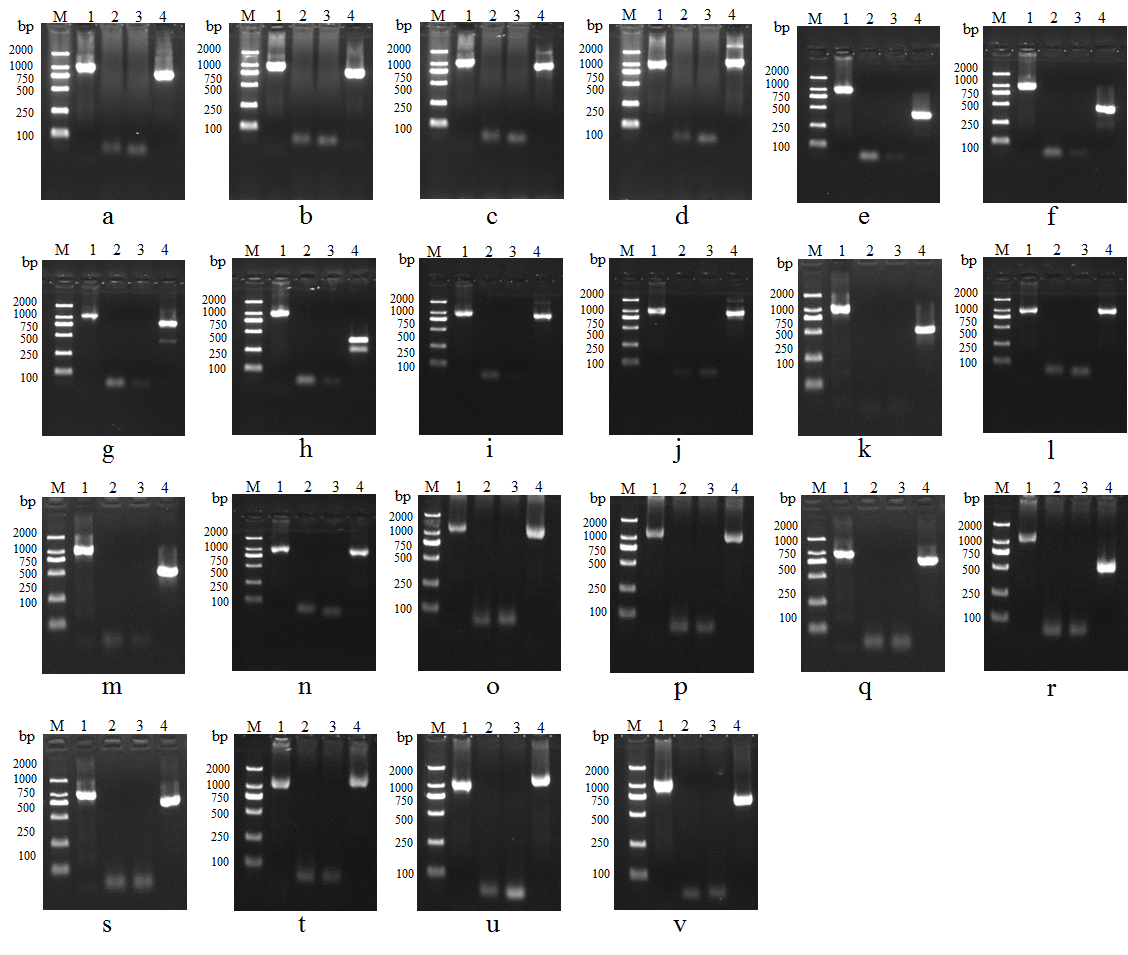

Supplement: S4 Fig — a-v: Representative PCR products for B. microti, B. divergens, B. duncani, B. venatorum, P. vivax, P. falciparum, P. knowlesi, P. malariae, P. ovale, L. donovani, L. gerbilli (18S rDNA gene), L. gerbilli (fumarate hydratase gene), L. tropica (18S rDNA gene), L. tropica (mspC gene), L. infantum, L. aethiopica, T. b. rhodesiense (18S rDNA gene), T. b. rhodesiense (lysosomal/endosomal membrane protein p67 gene), T. b. gambiense (18S rDNA gene), T. b. gambiense (hypothetical protein gene), T. cruzi and T. gondii. M: Marker; 1: positive control; 2: negative; 3: blood of health human; 4: reference sample. (TIF) [file pntd.0005160.s004.tif]

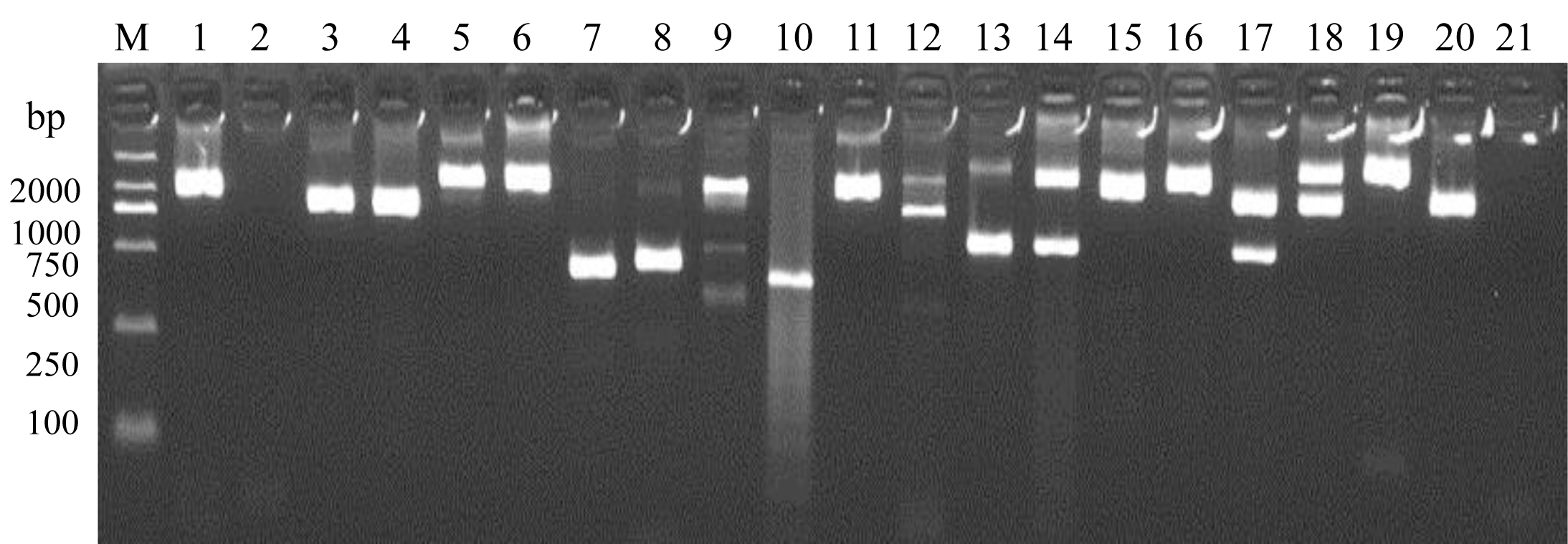

Supplement: S5 Fig — M: Marker; 1: positive control; 2: negative; 3: B. microti; 4: B. divergens; 5: B. duncani; 6: B. venatorum; 7: P. vivax; 8: P. falciparum; 9: P. knowlesi; 10: P. malariae; 11: P. ovale; 12: L. donovani; 13: L. gerbilli; 14: L. tropica; 15: L. infantum; 16: L. aethiopica; 17: T. b. rhodesiense; 18: T. b. gambiense; 19: T. cruzi; 20: T. gondii; 21: healthy adult. (TIF) [file pntd.0005160.s005.tif]

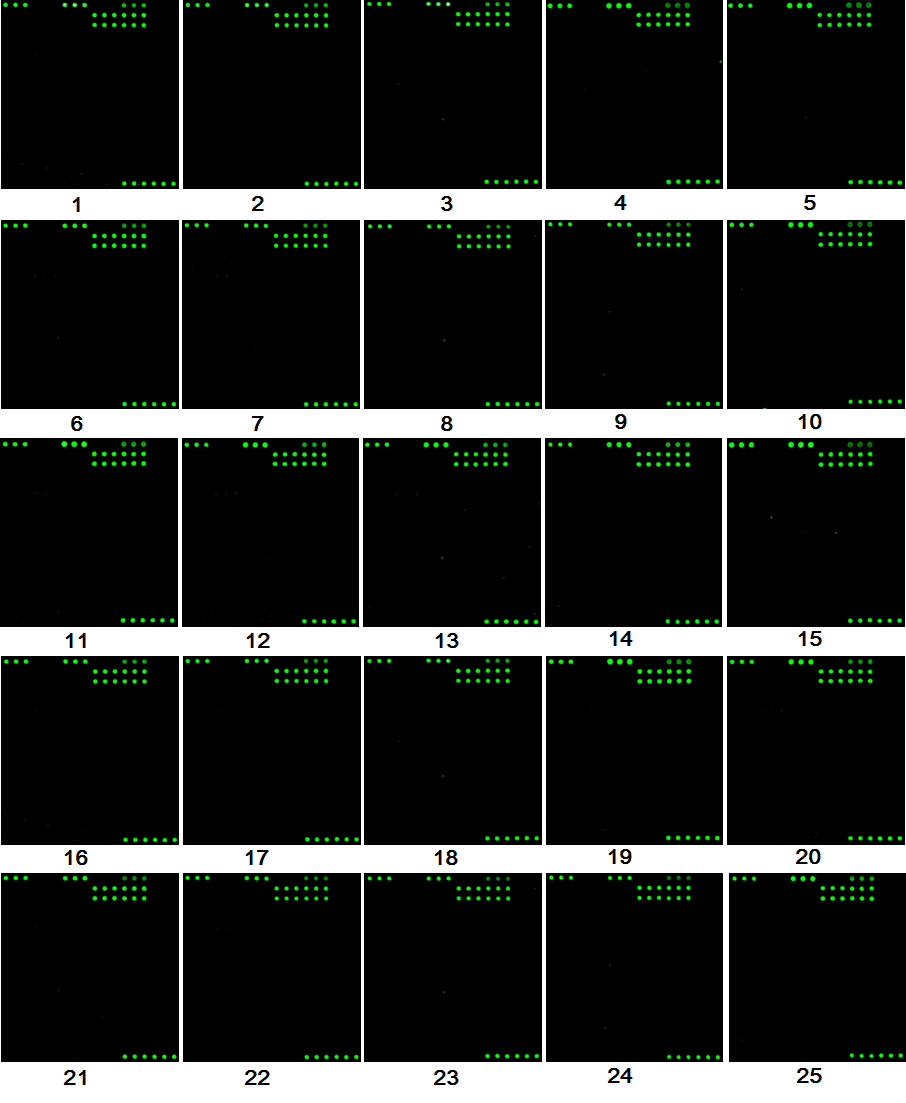

Supplement: S6 Fig — 1–5: 5 times repeatability of DNA microarray of B. microti with the array of 20150925 batche; 6–10: 5 times repeatability of DNA microarray of B. microti with the array of 20151026 batche; 11–15: 5 times repeatability of DNA microarray of B. microti with the array of 20151125 batche; 16–20: 5 times repeatability of DNA microarray of B. microti with the array of 20151224 batche; 21–25: 5 times repeatability of DNA microarray of B. microti with the array of 20160125 batche. (TIF) [file pntd.0005160.s006.tif]

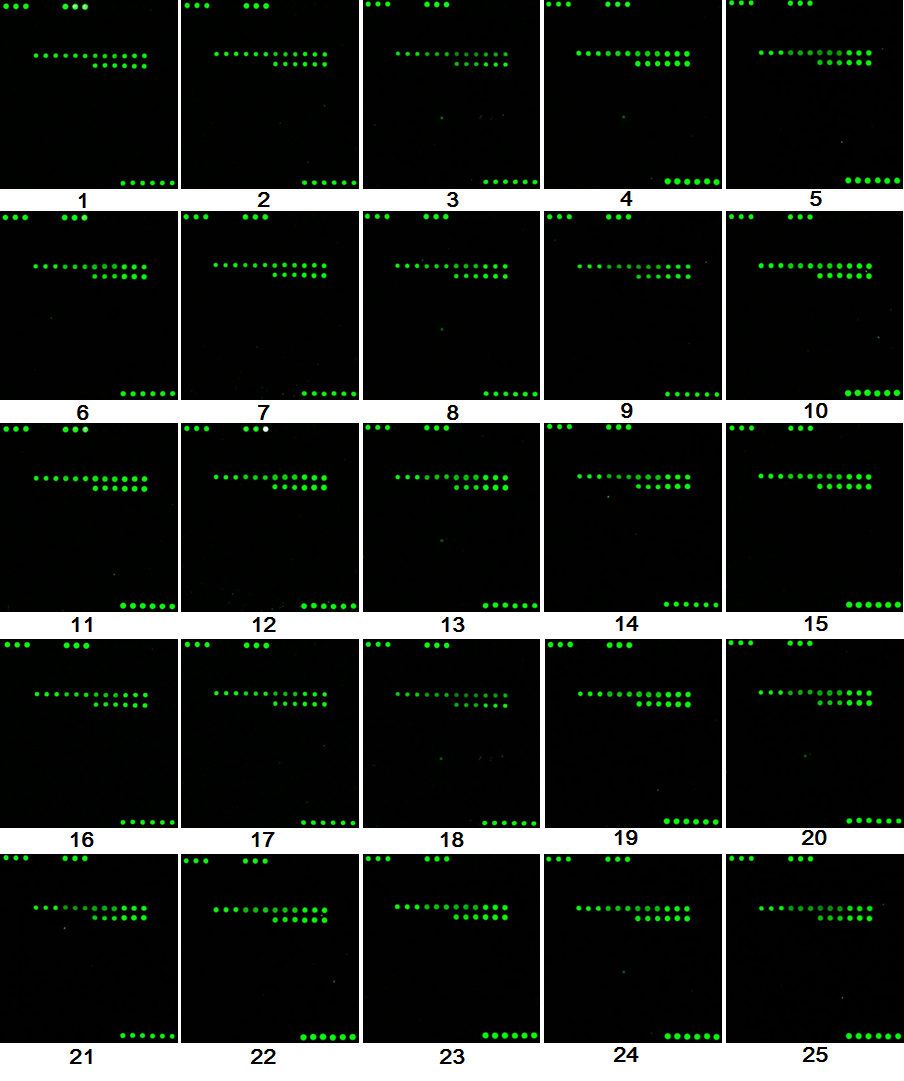

Supplement: S7 Fig — 1–5: 5 times repeatability of DNA microarray of P. falciparum with the array of 20150925 batche; 6–10: 5 times repeatability of DNA microarray of P. falciparum with the array of 20151026 batche; 11–15: 5 times repeatability of DNA microarray of P. falciparum with the array of 20151125 batche; 16–20: 5 times repeatability of DNA microarray of P. falciparum with the array of 20151224 batche; 21–25: 5 times repeatability of DNA microarray of P. falciparum with the array of 20160125 batche. (TIF) [file pntd.0005160.s007.tif]
