# Supplementary material for: DNA Microarray Detection of 18 Important Human Blood Protozoan Species
Source: PLoS Negl Trop Dis. 2016 Dec 2;10(12):e0005160. doi: 10.1371/journal.pntd.0005160 (PMC5135439; doi:10.1371/journal.pntd.0005160)
Supplement: S3 Fig — (PDF) [file pntd.0005160.s003.pdf]

中国疾病预防控制中心寄生虫病预防控制所

中国疾病预防控制中心寄生虫病预防控制所

（世界卫生组织疟疾、血吸虫病、丝虫病合作中心）

伦 理 审 查 委 员 会

NATIONAL INSTITUTE OF PARASITIC DISEASES (NIPD), CHINESE CENTER FOR DISEASE CONTROL AND PREVENTION (WHO COLLABORATING CENTRE FOR MALARIA, SCHISTOSOMIASIS AND FILARIASIS)

ETHICAL REVIEW COMMITTEE

Approval Notice

PRINCIPAL INVESTIGATOR OF PROJECT: Zhou Xiaonong

TITLE OF PROJECT: National S & T Major Program (Grant No. 2012ZX10004-220)

INSTITUTE: National Institute of Parasitic Diseases, China CDC

The Ethical Review Committee of National Institute of Parasitic Diseases, Chinese Center for Disease Control and Prevention has reviewed the proposal of "National S & T Major Program (Grant No. 2012ZX10004-220)". This project only deals with collecting elementary information about social, environmental, household and individual characteristics, collecting biological materials including feces and blood samples of human beings. It is recognized that all those methods for survey and samples collecting will be well accepted by the local people, the right and the welfare of the subject are adequately protected and the potential risks are outweighed by potential benefits.

SIGNATURE: Tang lh

Tang linhua, MD, Professor

Chair, Ethical Review Committee, NIPD, China CDC

August 26, 2012

地址：上海市瑞金二路207号
邮编：200025
电话 总机：21-64377008
直线：21-64376308
传真：86-21-64332670

IPD-2012-5

中国疾病预防控制中心寄生虫病预防控制所

实验动物福利伦理审查申请表

National Institute of Parasite Diseases Chinese Center for Disease Control and Prevention
The Application Form for Animal Experimental Welfare & Ethical Inspection

一、 基本信息 (Basic Information)

实验项目名称<br>Name of Research Project	重要寄生虫病监测技术研究(2012ZX10004-220)<br>The research of important parasitic disease monitoring technology (2012ZX10004-220)		
项目负责人姓名<br>Name of Applicant	周晓农<br>Zhou Xiaonong	职称/职务<br>Professional title	研究员<br>Professor
电话/传真<br>Telephone/FAX	86-21-64378058	电子邮件<br>E-mail	ipdzhouxn@sh163.net
部门名称<br>Name of Department	健教中心、重点实验室<br>Health Education Center, Key Laboratory of Parasite and Vector,		
项目的意义及必要性<br>Importance of Research Project	本研究进行血吸虫、疟原虫、巴贝虫等寄生虫动物感染模型的建立，从而加速寄生虫病监测研究工作进程<br>The establishment of animals model infected with *schistosoma japomicum, plasmodium* , *Babesia* etc. In order to promote the study of important parasitic disease monitoring technology.		
实验周期<br>Experimental periods	自：2012 年 1 月 1 日 至：2015 年 12 月 31 日		

二、 直接进行动物实验的人员资料 (Information of Personnel responsible for animal care & use):

姓名<br>Name	性别<br>Gender	职称/职务<br>Professional title	岗位证书编号<br>No. of Certification	实验动物经验<br>Experience of Animal Experiment
陈韶红<br>Chen Shaohong	女<br>F	研究员<br>Researcher		■有 Experienced<br>□需要带教 Under the supervision of other Experienced personnel
蔡玉春<br>Cai Yuchun	女<br>F	研究实习员<br>Research Assistant		■有 Experienced<br>□需要带教 Under the supervision of other Experienced personnel
徐斌<br>Xu Bin	女<br>F	副研究员<br>associate Researcher	11073845	■有 Experienced<br>□需要带教 Under the supervision of other Experienced personnel
胡媛<br>Hu Yuan	女<br>F	副研究员<br>associate Researcher	11073846	■有 Experienced<br>□需要带教 Under the supervision of other Experienced personnel
秦志强<br>Qin	女<br>F	副研究员<br>associate		■有 Experienced<br>□需要带教 Under the supervision of other

Zhiqiang		Researcher		Experienced personnel

三、 **实验动物信息 (Laboratory Animals Information):**

动物品种/品系 Animal Species	年龄/体重 Age/Weight	数量（Quantity）		供应单位 Supplier
		♀	♂	
1. KM mice	18-22	300	0	SLAC,Shanghai
2. BALB/c mice	18-22	400	0	SLAC,Shanghai
3. NOD-SCID mice	18-22	200	0	SLAC,Shanghai

四、 **动物饲养(Animal breeding):**

■由动物中心专人负责（Charging by special person in the animal center）;

☐由实验室人员负责，负责人姓名（Charging by lab staff, name of principle）:__________________

五、 **动物实验的主要内容及病原（The Main Content and pathogen of the Animal Experiment）:**

接种血吸虫、巴贝虫等寄生虫

Inoculate intraperitoneally into each mice with *schistosoma japomicum, plasmodium* , *Babesia* etc.

六、 **简述下列实验步骤(Describe the following experiment step in brief):**

(1) 使用麻醉药品名称及麻醉方式（Name and methods of Anaesthesia）

乙醚 diethyl ether 或者 or CO_2

注：使用超过一种实验动物时，须分别填报每种动物所使用的药品剂量（In the occasion that more than one sort of animal is needed, Please fill out the leechdom dosage that each sort animal is used.）。

无须麻醉原因（The reason of no Anaesthesia）: ________________________

(2) 若动物需长时间保定超过四小时，请说明所用器械与方法（If animal should be implemented restraint for more than 4 hours, Please describe the Methods of restraint）:

保定器 noose

七、 **请说明实验结束后动物的处置方式(Please explain the disposal methods of animal after experiment):**

(1) 实验后动物疼痛处理及照顾办法(The tendance methods for animal after experiment):

实验中不涉及此步骤 No this step

(2) 安乐死的方法(Methods of euthanasia):

麻醉后颈椎脱臼处死法 Cervical dislocation after naesthesia

(3) 实验动物尸体处理办法(Disposal of carcase):

■委托动物中心处理（Consign it to animal center） ☐其他（Others）:__________________

本人保证以上所填数据完全属实（I assure that the above date is true.）

项目负责人签字(Signature of Applicant): [signature]

填报日期（Date）2012 年（YYYY）8 月（MM）5 日（DD）

审查结果（Result of Inspection）

☑通过 Approve

☐修正通过 Approve in terms of Modification

修正意见：<br>Opinions	

☐不通过 Disapprove

建议事项：<br>suggestions	

中国疾病预防控制中心寄生虫病预防控制所实验动物福利伦理审查委员会

Laboratory Animal Welfare & Ethics Committee (LAWEC)，National Institute of Parasitic Diseases of China CDC

主席（或授权人）签章（Chairman' s Signature)：

[signature]

日期（Date）：2012 年（YYYY）9 月（MM）13 日（DD）
