# Supplementary material for: DNA Microarray Detection of 18 Important Human Blood Protozoan Species
Source: PLoS Negl Trop Dis. 2016 Dec 2;10(12):e0005160. doi: 10.1371/journal.pntd.0005160 (PMC5135439; doi:10.1371/journal.pntd.0005160)
Supplement: S1 Table — (DOCX) [file pntd.0005160.s008.docx]

**Table S1.** Reference blood protozoa samples

| **NO.** | **Species** | | **Isolates** | **Origin** | **Host** | **Diagnosis method** |
| --- | --- | --- | --- | --- | --- | --- |
| 1 | *Babesia microti* | | ATCC PRA-99TM | USA | field mouse | reference isolate |
| 2 | *Babesia divergens* | | Clinical reference | Heilongjiang, China | tick | PCR |
| 3 | *Babesia venatorum* | | Clinical reference | Heilongjiang, China | human | morphology+PCR |
| 4 | *Babesia duncani* | | Clinical reference | USA | tick | PCR |
| 5 | *Plasmodium vivax* | | Clinical reference | Yunnan,China | human | RDT+morphology+PCR |
| 6 | *Plasmodium falciparum* | | 3D7 |  |  | reference isolate |
| 7 | *Plasmodium knowlesi* | | Clinical reference | Yunnan,China | human | morphology+PCR |
| 8 | *Plasmodium malariae* | | Clinical reference | Yunnan,China | human | RDT+morphology+PCR |
| 9 | *Plasmodium ovale* | | Clinical reference | Yunnan,China | human | RDT+morphology+PCR |
| 10 | *Leishmania gerbilli* | | MRHO/CN/60/GERBILLI | Gansu, China | sand rat | WHO reference isolate |
| 11 | *Leishmania donovani* | | MHOM/IN/80/DD8 | India | human | WHO reference isolate |
| 12 | *Leishmania infantum* | | MHOM/CN/86/SC6 | Sichuan, China | human | RDT+morphology+PCR |
| 13 | *Leishmania tropica* | | MHOM/SU/74/K27 | Azerbaijan | human | WHO reference isolate |
| 14 | *Leishmania aethiopica* | | MHOM/ET/72/L100 | Ethiopia | human | WHO reference isolate |
| 15 | *Trypanosoma cruzi* | | Clinical reference | Brazil | human | morphology+PCR |
| 16 | *Trypanosoma brucei rhodesiense* | | YTAT 1.1 PF | Africa | rat | reference isolate |
| 17 | *Trypanosoma brucei gambiense* | | Clinical reference | Gabon | human | RDT+morphology+PCR |
| 18 | *Toxoplasma gondii* | TgCatBr5 | | Brazil | cat | reference isolate |
