# Supplementary material for: DNA Microarray Detection of 18 Important Human Blood Protozoan Species
Source: PLoS Negl Trop Dis. 2016 Dec 2;10(12):e0005160. doi: 10.1371/journal.pntd.0005160 (PMC5135439; doi:10.1371/journal.pntd.0005160)
Supplement: S2 Table — (DOCX) [file pntd.0005160.s009.docx]

**Table S2.** Protozoa clinical isolates of reference blood

| **NO.** | **Species** | **Origin** | **Host** | **Type** | **Diagnosis method** |
| --- | --- | --- | --- | --- | --- |
| Bm001 | *B. microti* | Yunnan,China | human | blood | PCR |
| Bm002 | *B. microti* | Yunnan,China | human | blood | PCR |
| Bm003 | *B. microti* | Yunnan,China | human | blood | PCR |
| Bm004 | *B. microti* | Shanghai,China | human | blood | morphology+PCR |
| Bd001 | *B. venatorum* | Heilongjiang, China | human | blood | morphology+PCR |
| Bd002 | *B. venatorum* | Heilongjiang, China | tick | whole | PCR |
| Bd003 | *B. venatorum* | Heilongjiang, China | tick | whole | PCR |
| Pv001 | *P. vivax* | Yunnan,China | human | blood | RDT+morphology+PCR |
| Pv002 | *P. vivax* | Yunnan,China | human | blood | RDT+morphology+PCR |
| Pv003 | *P. vivax* | Yunnan,China | human | blood | RDT+morphology+PCR |
| Pv004 | *P. vivax* | Yunnan,China | human | blood | RDT+morphology+PCR |
| Pv005 | *P. vivax* | Yunnan,China | human | blood | RDT+morphology+PCR |
| Pv006 | *P. vivax* | Yunnan,China | human | blood | RDT+morphology+PCR |
| Pv007 | *P. vivax* | Yunnan,China | human | blood | RDT+morphology+PCR |
| Pv008 | *P. vivax* | Yunnan,China | human | blood | RDT+morphology+PCR |
| Pv009 | *P. vivax* | Yunnan,China | human | blood | RDT+morphology+PCR |
| Pv010 | *P. vivax* | Yunnan,China | human | blood | RDT+morphology+PCR |
| Pv011 | *P. vivax* | Yunnan,China | human | blood | RDT+morphology+PCR |
| Pv012 | *P. vivax* | Yunnan,China | human | blood | RDT+morphology+PCR |
| Pv013 | *P. vivax* | Yunnan,China | human | blood | RDT+morphology+PCR |
| Pv014 | *P. vivax* | Yunnan,China | human | blood | RDT+morphology+PCR |
| Pv015 | *P. vivax* | Yunnan,China | human | blood | RDT+morphology+PCR |
| Pv016 | *P. vivax* | Yunnan,China | human | blood | RDT+morphology+PCR |
| Pv017 | *P. vivax* | Yunnan,China | human | blood | RDT+morphology+PCR |
| Pv018 | *P. vivax* | Yunnan,China | human | blood | RDT+morphology+PCR |
| Pv019 | *P. vivax* | Yunnan,China | human | blood | RDT+morphology+PCR |
| Pv020 | *P. vivax* | Yunnan,China | human | blood | RDT+morphology+PCR |
| Pv021 | *P. vivax* | Yunnan,China | human | blood | RDT+morphology+PCR |
| Pv022 | *P. vivax* | Yunnan,China | human | blood | RDT+morphology+PCR |
| Pv023 | *P. vivax* | Yunnan,China | human | blood | RDT+morphology+PCR |
| Pv024 | *P. vivax* | Yunnan,China | human | blood | RDT+morphology+PCR |
| Pv025 | *P. vivax* | Yunnan,China | human | blood | RDT+morphology+PCR |
| Pv026 | *P. vivax* | Yunnan,China | human | blood | RDT+morphology+PCR |
| Pv027 | *P. vivax* | Yunnan,China | human | blood | RDT+morphology+PCR |
| Pv028 | *P. vivax* | Yunnan,China | human | blood | RDT+morphology+PCR |
| Pv029 | *P. vivax* | Yunnan,China | human | blood | RDT+morphology+PCR |
| Pv030 | *P. vivax* | Yunnan,China | human | blood | RDT+morphology+PCR |
| Pv031 | *P. vivax* | Yunnan,China | human | blood | RDT+morphology+PCR |
| Pv032 | *P. vivax* | Yunnan,China | human | blood | RDT+morphology+PCR |
| Pv033 | *P. vivax* | Yunnan,China | human | blood | RDT+morphology+PCR |
| Pv034 | *P. vivax* | Yunnan,China | human | blood | RDT+morphology+PCR |
| Pv035 | *P. vivax* | Yunnan,China | human | blood | RDT+morphology+PCR |
| Pv036 | *P. vivax* | Yunnan,China | human | blood | RDT+morphology+PCR |
| Pv037 | *P. vivax* | Yunnan,China | human | blood | RDT+morphology+PCR |
| Pv038 | *P. vivax* | Yunnan,China | human | blood | RDT+morphology+PCR |
| Pv039 | *P. vivax* | Yunnan,China | human | blood | RDT+morphology+PCR |
| Pv040 | *P. vivax* | Yunnan,China | human | blood | RDT+morphology+PCR |
| Pv041 | *P. vivax* | Yunnan,China | human | blood | RDT+morphology+PCR |
| Pv042 | *P. vivax* | Yunnan,China | human | blood | RDT+morphology+PCR |
| Pv043 | *P. vivax* | Yunnan,China | human | blood | RDT+morphology+PCR |
| Pv044 | *P. vivax* | Yunnan,China | human | blood | RDT+morphology+PCR |
| Pv045 | *P. vivax* | Yunnan,China | human | blood | RDT+morphology+PCR |
| Pv046 | *P. vivax* | Yunnan,China | human | blood | RDT+morphology+PCR |
| Pv047 | *P. vivax* | Yunnan,China | human | blood | RDT+morphology+PCR |
| Pv048 | *P. vivax* | Yunnan,China | human | blood | RDT+morphology+PCR |
| Pv049 | *P. vivax* | Yunnan,China | human | blood | RDT+morphology+PCR |
| Pv050 | *P. vivax* | Yunnan,China | human | blood | RDT+morphology+PCR |
| Pv051 | *P. vivax* | Yunnan,China | human | blood | RDT+morphology+PCR |
| Pv052 | *P. vivax* | Yunnan,China | human | blood | RDT+morphology+PCR |
| Pv053 | *P. vivax* | Yunnan,China | human | blood | RDT+morphology+PCR |
| Pv054 | *P. vivax* | Yunnan,China | human | blood | RDT+morphology+PCR |
| Pv055 | *P. vivax* | Yunnan,China | human | blood | RDT+morphology+PCR |
| Pv056 | *P. vivax* | Yunnan,China | human | blood | RDT+morphology+PCR |
| Pv057 | *P. vivax* | Yunnan,China | human | blood | RDT+morphology+PCR |
| Pv058 | *P. vivax* | Yunnan,China | human | blood | RDT+morphology+PCR |
| Pv059 | *P. vivax* | Yunnan,China | human | blood | RDT+morphology+PCR |
| Pv060 | *P. vivax* | Yunnan,China | human | blood | RDT+morphology+PCR |
| Pv061 | *P. vivax* | Yunnan,China | human | blood | RDT+morphology+PCR |
| Pv062 | *P. vivax* | Yunnan,China | human | blood | RDT+morphology+PCR |
| Pv063 | *P. vivax* | Yunnan,China | human | blood | RDT+morphology+PCR |
| Pv064 | *P. vivax* | Yunnan,China | human | blood | RDT+morphology+PCR |
| Pv065 | *P. vivax* | Yunnan,China | human | blood | RDT+morphology+PCR |
| Pv066 | *P. vivax* | Yunnan,China | human | blood | RDT+morphology+PCR |
| Pv067 | *P. vivax* | Yunnan,China | human | blood | RDT+morphology+PCR |
| Pv068 | *P. vivax* | Yunnan,China | human | blood | RDT+morphology+PCR |
| Pv069 | *P. vivax* | Yunnan,China | human | blood | RDT+morphology+PCR |
| Pv070 | *P. vivax* | Yunnan,China | human | blood | RDT+morphology+PCR |
| Pv071 | *P. vivax* | Yunnan,China | human | blood | RDT+morphology+PCR |
| Pv072 | *P. vivax* | Yunnan,China | human | blood | RDT+morphology+PCR |
| Pv073 | *P. vivax* | Yunnan,China | human | blood | RDT+morphology+PCR |
| Pv074 | *P. vivax* | Yunnan,China | human | blood | RDT+morphology+PCR |
| Pv075 | *P. vivax* | Yunnan,China | human | blood | RDT+morphology+PCR |
| Pv076 | *P. vivax* | Yunnan,China | human | blood | RDT+morphology+PCR |
| Pv077 | *P. vivax* | Yunnan,China | human | blood | RDT+morphology+PCR |
| Pv078 | *P. vivax* | Yunnan,China | human | blood | RDT+morphology+PCR |
| Pv079 | *P. vivax* | Yunnan,China | human | blood | RDT+morphology+PCR |
| Pv080 | *P. vivax* | Yunnan,China | human | blood | RDT+morphology+PCR |
| Pv081 | *P. vivax* | Yunnan,China | human | blood | RDT+morphology+PCR |
| Pv082 | *P. vivax* | Yunnan,China | human | blood | RDT+morphology+PCR |
| Pv083 | *P. vivax* | Yunnan,China | human | blood | RDT+morphology+PCR |
| Pv084 | *P. vivax* | Yunnan,China | human | blood | RDT+morphology+PCR |
| Pv085 | *P. vivax* | Yunnan,China | human | blood | RDT+morphology+PCR |
| Pv086 | *P. vivax* | Yunnan,China | human | blood | RDT+morphology+PCR |
| Pv087 | *P. vivax* | Yunnan,China | human | blood | RDT+morphology+PCR |
| Pv088 | *P. vivax* | Yunnan,China | human | blood | RDT+morphology+PCR |
| Pv089 | *P. vivax* | Yunnan,China | human | blood | RDT+morphology+PCR |
| Pv090 | *P. vivax* | Yunnan,China | human | blood | RDT+morphology+PCR |
| Pv091 | *P. vivax* | Yunnan,China | human | blood | RDT+morphology+PCR |
| Pv092 | *P. vivax* | Yunnan,China | human | blood | RDT+morphology+PCR |
| Pv093 | *P. vivax* | Yunnan,China | human | blood | RDT+morphology+PCR |
| Pv094 | *P. vivax* | Yunnan,China | human | blood | RDT+morphology+PCR |
| Pv095 | *P. vivax* | Yunnan,China | human | blood | RDT+morphology+PCR |
| Pv096 | *P. vivax* | Yunnan,China | human | blood | RDT+morphology+PCR |
| Pv097 | *P. vivax* | Yunnan,China | human | blood | RDT+morphology+PCR |
| Pv098 | *P. vivax* | Yunnan,China | human | blood | RDT+morphology+PCR |
| Pv099 | *P. vivax* | Yunnan,China | human | blood | RDT+morphology+PCR |
| Pv100 | *P. vivax* | Yunnan,China | human | blood | RDT+morphology+PCR |
| Pv101 | *P. vivax* | Yunnan,China | human | blood | RDT+morphology+PCR |
| Pv102 | *P. vivax* | Yunnan,China | human | blood | RDT+morphology+PCR |
| Pv103 | *P. vivax* | Yunnan,China | human | blood | RDT+morphology+PCR |
| Pv104 | *P. vivax* | Yunnan,China | human | blood | RDT+morphology+PCR |
| Pv105 | *P. vivax* | Yunnan,China | human | blood | RDT+morphology+PCR |
| Pv106 | *P. vivax* | Hainan,China | human | blood | RDT+morphology+PCR |
| Pv107 | *P. vivax* | Hainan,China | human | blood | RDT+morphology+PCR |
| Pv108 | *P. vivax* | Hainan,China | human | blood | RDT+morphology+PCR |
| Pv109 | *P. vivax* | Hainan,China | human | blood | RDT+morphology+PCR |
| Pv110 | *P. vivax* | Hainan,China | human | blood | RDT+morphology+PCR |
| Pv111 | *P. vivax* | Hainan,China | human | blood | RDT+morphology+PCR |
| Pv112 | *P. vivax* | Hainan,China | human | blood | RDT+morphology+PCR |
| Pv113 | *P. vivax* | Hainan,China | human | blood | RDT+morphology+PCR |
| Pv114 | *P. vivax* | Hainan,China | human | blood | RDT+morphology+PCR |
| Pv115 | *P. vivax* | Hainan,China | human | blood | RDT+morphology+PCR |
| Pv116 | *P. vivax* | Hainan,China | human | blood | RDT+morphology+PCR |
| Pv117 | *P. vivax* | Hainan,China | human | blood | RDT+morphology+PCR |
| Pv118 | *P. vivax* | Hainan,China | human | blood | RDT+morphology+PCR |
| Pv119 | *P. vivax* | Hainan,China | human | blood | RDT+morphology+PCR |
| Pv120 | *P. vivax* | Hainan,China | human | blood | RDT+morphology+PCR |
| Pv121 | *P. vivax* | Hainan,China | human | blood | RDT+morphology+PCR |
| Pv122 | *P. vivax* | Hainan,China | human | blood | RDT+morphology+PCR |
| Pv123 | *P. vivax* | Hainan,China | human | blood | RDT+morphology+PCR |
| Pv124 | *P. vivax* | Hainan,China | human | blood | RDT+morphology+PCR |
| Pv125 | *P. vivax* | Hainan,China | human | blood | RDT+morphology+PCR |
| Pv126 | *P. vivax* | Hainan,China | human | blood | RDT+morphology+PCR |
| Pv127 | *P. vivax* | Hainan,China | human | blood | RDT+morphology+PCR |
| Pv128 | *P. vivax* | Hainan,China | human | blood | RDT+morphology+PCR |
| Pv129 | *P. vivax* | Hainan,China | human | blood | RDT+morphology+PCR |
| Pv130 | *P. vivax* | Hainan,China | human | blood | RDT+morphology+PCR |
| Pv131 | *P. vivax* | Hainan,China | human | blood | RDT+morphology+PCR |
| Pv132 | *P. vivax* | Hainan,China | human | blood | RDT+morphology+PCR |
| Pv133 | *P. vivax* | Hainan,China | human | blood | RDT+morphology+PCR |
| Pv134 | *P. vivax* | Hainan,China | human | blood | RDT+morphology+PCR |
| Pv135 | *P. vivax* | Hainan,China | human | blood | RDT+morphology+PCR |
| Pf001 | *P. falciparum* | Yunnan,China | human | blood | RDT+morphology+PCR |
| Pf002 | *P. falciparum* | Yunnan,China | human | blood | RDT+morphology+PCR |
| Pf003 | *P. falciparum* | Yunnan,China | human | blood | RDT+morphology+PCR |
| Pf004 | *P. falciparum* | Yunnan,China | human | blood | RDT+morphology+PCR |
| Pf005 | *P. falciparum* | Yunnan,China | human | blood | RDT+morphology+PCR |
| Pf006 | *P. falciparum* | Yunnan,China | human | blood | RDT+morphology+PCR |
| Pf007 | *P. falciparum* | Yunnan,China | human | blood | RDT+morphology+PCR |
| Pf008 | *P. falciparum* | Yunnan,China | human | blood | RDT+morphology+PCR |
| Pf009 | *P. falciparum* | Yunnan,China | human | blood | RDT+morphology+PCR |
| Pf010 | *P. falciparum* | Yunnan,China | human | blood | RDT+morphology+PCR |
| Pf011 | *P. falciparum* | Yunnan,China | human | blood | RDT+morphology+PCR |
| Pf012 | *P. falciparum* | Yunnan,China | human | blood | RDT+morphology+PCR |
| Pf013 | *P. falciparum* | Yunnan,China | human | blood | RDT+morphology+PCR |
| Pf014 | *P. falciparum* | Yunnan,China | human | blood | RDT+morphology+PCR |
| Pf015 | *P. falciparum* | Yunnan,China | human | blood | RDT+morphology+PCR |
| Pf016 | *P. falciparum* | Yunnan,China | human | blood | RDT+morphology+PCR |
| Pf017 | *P. falciparum* | Yunnan,China | human | blood | RDT+morphology+PCR |
| Pf018 | *P. falciparum* | Yunnan,China | human | blood | RDT+morphology+PCR |
| Pf019 | *P. falciparum* | Yunnan,China | human | blood | RDT+morphology+PCR |
| Pf020 | *P. falciparum* | Yunnan,China | human | blood | RDT+morphology+PCR |
| Pf021 | *P. falciparum* | Yunnan,China | human | blood | RDT+morphology+PCR |
| Pf022 | *P. falciparum* | Yunnan,China | human | blood | RDT+morphology+PCR |
| Pf023 | *P. falciparum* | Yunnan,China | human | blood | RDT+morphology+PCR |
| Pf024 | *P. falciparum* | Yunnan,China | human | blood | RDT+morphology+PCR |
| Pf025 | *P. falciparum* | Yunnan,China | human | blood | RDT+morphology+PCR |
| Pf026 | *P. falciparum* | Yunnan,China | human | blood | RDT+morphology+PCR |
| Pf027 | *P. falciparum* | Yunnan,China | human | blood | RDT+morphology+PCR |
| Pf028 | *P. falciparum* | Yunnan,China | human | blood | RDT+morphology+PCR |
| Pf029 | *P. falciparum* | Yunnan,China | human | blood | RDT+morphology+PCR |
| Pf030 | *P. falciparum* | Yunnan,China | human | blood | RDT+morphology+PCR |
| Pf031 | *P. falciparum* | Yunnan,China | human | blood | RDT+morphology+PCR |
| Pf032 | *P. falciparum* | Yunnan,China | human | blood | RDT+morphology+PCR |
| Pf033 | *P. falciparum* | Yunnan,China | human | blood | RDT+morphology+PCR |
| Pf034 | *P. falciparum* | Yunnan,China | human | blood | RDT+morphology+PCR |
| Pf035 | *P. falciparum* | Yunnan,China | human | blood | RDT+morphology+PCR |
| Pf036 | *P. falciparum* | Yunnan,China | human | blood | RDT+morphology+PCR |
| Pf037 | *P. falciparum* | Yunnan,China | human | blood | RDT+morphology+PCR |
| Pf038 | *P. falciparum* | Yunnan,China | human | blood | RDT+morphology+PCR |
| Pf039 | *P. falciparum* | Yunnan,China | human | blood | RDT+morphology+PCR |
| Pf040 | *P. falciparum* | Yunnan,China | human | blood | RDT+morphology+PCR |
| Pf041 | *P. falciparum* | Yunnan,China | human | blood | RDT+morphology+PCR |
| Pf042 | *P. falciparum* | Yunnan,China | human | blood | RDT+morphology+PCR |
| Pf043 | *P. falciparum* | Yunnan,China | human | blood | RDT+morphology+PCR |
| Pf044 | *P. falciparum* | Yunnan,China | human | blood | RDT+morphology+PCR |
| Pf045 | *P. falciparum* | Yunnan,China | human | blood | RDT+morphology+PCR |
| Pf046 | *P. falciparum* | Yunnan,China | human | blood | RDT+morphology+PCR |
| Pf047 | *P. falciparum* | Yunnan,China | human | blood | RDT+morphology+PCR |
| Pf048 | *P. falciparum* | Yunnan,China | human | blood | RDT+morphology+PCR |
| Pf049 | *P. falciparum* | Yunnan,China | human | blood | RDT+morphology+PCR |
| Pf050 | *P. falciparum* | Yunnan,China | human | blood | RDT+morphology+PCR |
| Pf051 | *P. falciparum* | Yunnan,China | human | blood | RDT+morphology+PCR |
| Pf052 | *P. falciparum* | Yunnan,China | human | blood | RDT+morphology+PCR |
| Pf053 | *P. falciparum* | Yunnan,China | human | blood | RDT+morphology+PCR |
| Pf054 | *P. falciparum* | Yunnan,China | human | blood | RDT+morphology+PCR |
| Pf055 | *P. falciparum* | Yunnan,China | human | blood | RDT+morphology+PCR |
| Pf056 | *P. falciparum* | Yunnan,China | human | blood | RDT+morphology+PCR |
| Pf057 | *P. falciparum* | Yunnan,China | human | blood | RDT+morphology+PCR |
| Pf058 | *P. falciparum* | Yunnan,China | human | blood | RDT+morphology+PCR |
| Pf059 | *P. falciparum* | Yunnan,China | human | blood | RDT+morphology+PCR |
| Pf060 | *P. falciparum* | Yunnan,China | human | blood | RDT+morphology+PCR |
| Pf061 | *P. falciparum* | Yunnan,China | human | blood | RDT+morphology+PCR |
| Pf062 | *P. falciparum* | Yunnan,China | human | blood | RDT+morphology+PCR |
| Pf063 | *P. falciparum* | Yunnan,China | human | blood | RDT+morphology+PCR |
| Pf064 | *P. falciparum* | Yunnan,China | human | blood | RDT+morphology+PCR |
| Pf065 | *P. falciparum* | Yunnan,China | human | blood | RDT+morphology+PCR |
| Pf066 | *P. falciparum* | Yunnan,China | human | blood | RDT+morphology+PCR |
| Pf067 | *P. falciparum* | Yunnan,China | human | blood | RDT+morphology+PCR |
| Pf068 | *P. falciparum* | Yunnan,China | human | blood | RDT+morphology+PCR |
| Pf069 | *P. falciparum* | Yunnan,China | human | blood | RDT+morphology+PCR |
| Pf070 | *P. falciparum* | Yunnan,China | human | blood | RDT+morphology+PCR |
| Pf071 | *P. falciparum* | Yunnan,China | human | blood | RDT+morphology+PCR |
| Pf072 | *P. falciparum* | Yunnan,China | human | blood | RDT+morphology+PCR |
| Pf073 | *P. falciparum* | Yunnan,China | human | blood | RDT+morphology+PCR |
| Pf074 | *P. falciparum* | Yunnan,China | human | blood | RDT+morphology+PCR |
| Pf075 | *P. falciparum* | Yunnan,China | human | blood | RDT+morphology+PCR |
| Pf076 | *P. falciparum* | Yunnan,China | human | blood | RDT+morphology+PCR |
| Pf077 | *P. falciparum* | Yunnan,China | human | blood | RDT+morphology+PCR |
| Pf078 | *P. falciparum* | Yunnan,China | human | blood | RDT+morphology+PCR |
| Pf079 | *P. falciparum* | Yunnan,China | human | blood | RDT+morphology+PCR |
| Pf080 | *P. falciparum* | Yunnan,China | human | blood | RDT+morphology+PCR |
| Pf081 | *P. falciparum* | Yunnan,China | human | blood | RDT+morphology+PCR |
| Pf082 | *P. falciparum* | Yunnan,China | human | blood | RDT+morphology+PCR |
| Pf083 | *P. falciparum* | Yunnan,China | human | blood | RDT+morphology+PCR |
| Pf084 | *P. falciparum* | Yunnan,China | human | blood | RDT+morphology+PCR |
| Pf085 | *P. falciparum* | Yunnan,China | human | blood | RDT+morphology+PCR |
| Pf086 | *P. falciparum* | Yunnan,China | human | blood | RDT+morphology+PCR |
| Pf087 | *P. falciparum* | Yunnan,China | human | blood | RDT+morphology+PCR |
| Pf088 | *P. falciparum* | Yunnan,China | human | blood | RDT+morphology+PCR |
| Pf089 | *P. falciparum* | Yunnan,China | human | blood | RDT+morphology+PCR |
| Pf090 | *P. falciparum* | Yunnan,China | human | blood | RDT+morphology+PCR |
| Pf091 | *P. falciparum* | Yunnan,China | human | blood | RDT+morphology+PCR |
| Pf092 | *P. falciparum* | Yunnan,China | human | blood | RDT+morphology+PCR |
| Pf093 | *P. falciparum* | Yunnan,China | human | blood | RDT+morphology+PCR |
| Pf094 | *P. falciparum* | Yunnan,China | human | blood | RDT+morphology+PCR |
| Pf095 | *P. falciparum* | Yunnan,China | human | blood | RDT+morphology+PCR |
| Pf096 | *P. falciparum* | Yunnan,China | human | blood | RDT+morphology+PCR |
| Pf097 | *P. falciparum* | Yunnan,China | human | blood | RDT+morphology+PCR |
| Pf098 | *P. falciparum* | Yunnan,China | human | blood | RDT+morphology+PCR |
| Pf099 | *P. falciparum* | Yunnan,China | human | blood | RDT+morphology+PCR |
| Pf100 | *P. falciparum* | Yunnan,China | human | blood | RDT+morphology+PCR |
| Pf101 | *P. falciparum* | Yunnan,China | human | blood | RDT+morphology+PCR |
| Pf102 | *P. falciparum* | Yunnan,China | human | blood | RDT+morphology+PCR |
| Pf103 | *P. falciparum* | Yunnan,China | human | blood | RDT+morphology+PCR |
| Pf104 | *P. falciparum* | Yunnan,China | human | blood | RDT+morphology+PCR |
| Pf105 | *P. falciparum* | Yunnan,China | human | blood | RDT+morphology+PCR |
| Pf106 | *P. falciparum* | Yunnan,China | human | blood | RDT+morphology+PCR |
| Pf107 | *P. falciparum* | Yunnan,China | human | blood | RDT+morphology+PCR |
| Pf108 | *P. falciparum* | Yunnan,China | human | blood | RDT+morphology+PCR |
| Pf109 | *P. falciparum* | Yunnan,China | human | blood | RDT+morphology+PCR |
| Pf110 | *P. falciparum* | Yunnan,China | human | blood | RDT+morphology+PCR |
| Pf111 | *P. falciparum* | Yunnan,China | human | blood | RDT+morphology+PCR |
| Pf112 | *P. falciparum* | Yunnan,China | human | blood | RDT+morphology+PCR |
| Pf113 | *P. falciparum* | Yunnan,China | human | blood | RDT+morphology+PCR |
| Pf114 | *P. falciparum* | Yunnan,China | human | blood | RDT+morphology+PCR |
| Pf115 | *P. falciparum* | Yunnan,China | human | blood | RDT+morphology+PCR |
| Pf116 | *P. falciparum* | Yunnan,China | human | blood | RDT+morphology+PCR |
| Pf117 | *P. falciparum* | Yunnan,China | human | blood | RDT+morphology+PCR |
| Pf118 | *P. falciparum* | Yunnan,China | human | blood | RDT+morphology+PCR |
| Pf119 | *P. falciparum* | Yunnan,China | human | blood | RDT+morphology+PCR |
| Pf120 | *P. falciparum* | Yunnan,China | human | blood | RDT+morphology+PCR |
| Pf121 | *P. falciparum* | Yunnan,China | human | blood | RDT+morphology+PCR |
| Pf122 | *P. falciparum* | Yunnan,China | human | blood | RDT+morphology+PCR |
| Pf123 | *P. falciparum* | Yunnan,China | human | blood | RDT+morphology+PCR |
| Pf124 | *P. falciparum* | Yunnan,China | human | blood | RDT+morphology+PCR |
| Pf125 | *P. falciparum* | Yunnan,China | human | blood | RDT+morphology+PCR |
| Pf126 | *P. falciparum* | Yunnan,China | human | blood | RDT+morphology+PCR |
| Pf127 | *P. falciparum* | Yunnan,China | human | blood | RDT+morphology+PCR |
| Pf128 | *P. falciparum* | Yunnan,China | human | blood | RDT+morphology+PCR |
| Pf129 | *P. falciparum* | Yunnan,China | human | blood | RDT+morphology+PCR |
| Pf130 | *P. falciparum* | Yunnan,China | human | blood | RDT+morphology+PCR |
| Pf131 | *P. falciparum* | Yunnan,China | human | blood | RDT+morphology+PCR |
| Pf132 | *P. falciparum* | Yunnan,China | human | blood | RDT+morphology+PCR |
| Pf133 | *P. falciparum* | Hainan,China | human | blood | RDT+morphology+PCR |
| Pf134 | *P. falciparum* | Hainan,China | human | blood | RDT+morphology+PCR |
| Pf135 | *P. falciparum* | Hainan,China | human | blood | RDT+morphology+PCR |
| Pf136 | *P. falciparum* | Hainan,China | human | blood | RDT+morphology+PCR |
| Pf137 | *P. falciparum* | Hainan,China | human | blood | RDT+morphology+PCR |
| Pf138 | *P. falciparum* | Hainan,China | human | blood | RDT+morphology+PCR |
| Pf139 | *P. falciparum* | Hainan,China | human | blood | RDT+morphology+PCR |
| Pf140 | *P. falciparum* | Hainan,China | human | blood | RDT+morphology+PCR |
| Pf141 | *P. falciparum* | Hainan,China | human | blood | RDT+morphology+PCR |
| Pf142 | *P. falciparum* | Hainan,China | human | blood | RDT+morphology+PCR |
| Pf143 | *P. falciparum* | Hainan,China | human | blood | RDT+morphology+PCR |
| Pf144 | *P. falciparum* | Hainan,China | human | blood | RDT+morphology+PCR |
| Pf145 | *P. falciparum* | Hainan,China | human | blood | RDT+morphology+PCR |
| Pf146 | *P. falciparum* | Hainan,China | human | blood | RDT+morphology+PCR |
| Pf147 | *P. falciparum* | Hainan,China | human | blood | RDT+morphology+PCR |
| Pf148 | *P. falciparum* | Hainan,China | human | blood | RDT+morphology+PCR |
| Pf149 | *P. falciparum* | Hainan,China | human | blood | RDT+morphology+PCR |
| Pf150 | *P. falciparum* | Hainan,China | human | blood | RDT+morphology+PCR |
| Pf151 | *P. falciparum* | Hainan,China | human | blood | RDT+morphology+PCR |
| Pf152 | *P. falciparum* | Hainan,China | human | blood | RDT+morphology+PCR |
| Pf153 | *P. falciparum* | Hainan,China | human | blood | RDT+morphology+PCR |
| Pf154 | *P. falciparum* | Hainan,China | human | blood | RDT+morphology+PCR |
| Pf155 | *P. falciparum* | Hainan,China | human | blood | RDT+morphology+PCR |
| Pf156 | *P. falciparum* | Hainan,China | human | blood | RDT+morphology+PCR |
| Pf157 | *P. falciparum* | Hainan,China | human | blood | RDT+morphology+PCR |
| Pf158 | *P. falciparum* | Hainan,China | human | blood | RDT+morphology+PCR |
| Pf159 | *P. falciparum* | Hainan,China | human | blood | RDT+morphology+PCR |
| Pf160 | *P. falciparum* | Hainan,China | human | blood | RDT+morphology+PCR |
| Pf161 | *P. falciparum* | Hainan,China | human | blood | RDT+morphology+PCR |
| Pf162 | *P. falciparum* | Hainan,China | human | blood | RDT+morphology+PCR |
| Pf163 | *P. falciparum* | Hainan,China | human | blood | RDT+morphology+PCR |
| Pk001 | *P. knowlesi* | Yunnan,China | human | blood | morphology+PCR |
| Pk002 | *P. knowlesi* | Yunnan,China | human | blood | morphology+PCR |
| Pk003 | *P. knowlesi* | Yunnan,China | human | blood | morphology+PCR |
| Pk004 | *P. knowlesi* | Yunnan,China | human | blood | morphology+PCR |
| Pk005 | *P. knowlesi* | Yunnan,China | human | blood | morphology+PCR |
| Pk006 | *P. knowlesi* | Yunnan,China | human | blood | morphology+PCR |
| Pk007 | *P. knowlesi* | Yunnan,China | human | blood | morphology+PCR |
| Pk008 | *P. knowlesi* | Yunnan,China | human | blood | morphology+PCR |
| Pm001 | *P. malariae* | Yunnan,China | human | blood | RDT+morphology+PCR |
| Pm002 | *P. malariae* | Yunnan,China | human | blood | RDT+morphology+PCR |
| Pm003 | *P. malariae* | Yunnan,China | human | blood | RDT+morphology+PCR |
| Pm004 | *P. malariae* | Yunnan,China | human | blood | RDT+morphology+PCR |
| Pm005 | *P. malariae* | Yunnan,China | human | blood | RDT+morphology+PCR |
| Pm006 | *P. malariae* | Yunnan,China | human | blood | RDT+morphology+PCR |
| Pm007 | *P. malariae* | Yunnan,China | human | blood | RDT+morphology+PCR |
| Pm008 | *P. malariae* | Yunnan,China | human | blood | RDT+morphology+PCR |
| Pm009 | *P. malariae* | Yunnan,China | human | blood | RDT+morphology+PCR |
| Pm010 | *P. malariae* | Yunnan,China | human | blood | RDT+morphology+PCR |
| Pm011 | *P. malariae* | Yunnan,China | human | blood | RDT+morphology+PCR |
| Pm012 | *P. malariae* | Yunnan,China | human | blood | RDT+morphology+PCR |
| Pm013 | *P. malariae* | Yunnan,China | human | blood | RDT+morphology+PCR |
| Pm014 | *P. malariae* | Yunnan,China | human | blood | RDT+morphology+PCR |
| Pm015 | *P. malariae* | Yunnan,China | human | blood | RDT+morphology+PCR |
| Pm016 | *P. malariae* | Yunnan,China | human | blood | RDT+morphology+PCR |
| Pm017 | *P. malariae* | Hainan,China | human | blood | RDT+morphology+PCR |
| Pm018 | *P. malariae* | Hainan,China | human | blood | RDT+morphology+PCR |
| Pm019 | *P. malariae* | Hainan,China | human | blood | RDT+morphology+PCR |
| Pm020 | *P. malariae* | Hainan,China | human | blood | RDT+morphology+PCR |
| Pm021 | *P. malariae* | Hainan,China | human | blood | RDT+morphology+PCR |
| Pm022 | *P. malariae* | Hainan,China | human | blood | RDT+morphology+PCR |
| Pm023 | *P. malariae* | Hainan,China | human | blood | RDT+morphology+PCR |
| Pm024 | *P. malariae* | Hainan,China | human | blood | RDT+morphology+PCR |
| Pm025 | *P. malariae* | Hainan,China | human | blood | RDT+morphology+PCR |
| Po001 | *P.ovale* | Yunnan,China | human | blood | RDT+morphology+PCR |
| Po002 | *P.ovale* | Yunnan,China | human | blood | RDT+morphology+PCR |
| Po003 | *P.ovale* | Yunnan,China | human | blood | RDT+morphology+PCR |
| Po004 | *P.ovale* | Yunnan,China | human | blood | RDT+morphology+PCR |
| Po005 | *P.ovale* | Yunnan,China | human | blood | RDT+morphology+PCR |
| Po006 | *P.ovale* | Yunnan,China | human | blood | RDT+morphology+PCR |
| Po007 | *P.ovale* | Yunnan,China | human | blood | RDT+morphology+PCR |
| Po008 | *P.ovale* | Yunnan,China | human | blood | RDT+morphology+PCR |
| Po009 | *P.ovale* | Yunnan,China | human | blood | RDT+morphology+PCR |
| Po010 | *P.ovale* | Yunnan,China | human | blood | RDT+morphology+PCR |
| Po011 | *P.ovale* | Yunnan,China | human | blood | RDT+morphology+PCR |
| Po012 | *P.ovale* | Yunnan,China | human | blood | RDT+morphology+PCR |
| Po013 | *P.ovale* | Yunnan,China | human | blood | RDT+morphology+PCR |
| Po014 | *P.ovale* | Yunnan,China | human | blood | RDT+morphology+PCR |
| Po015 | *P.ovale* | Yunnan,China | human | blood | RDT+morphology+PCR |
| Po016 | *P.ovale* | Yunnan,China | human | blood | RDT+morphology+PCR |
| Po017 | *P.ovale* | Yunnan,China | human | blood | RDT+morphology+PCR |
| Po018 | *P.ovale* | Yunnan,China | human | blood | RDT+morphology+PCR |
| Po019 | *P.ovale* | Yunnan,China | human | blood | RDT+morphology+PCR |
| Po020 | *P.ovale* | Yunnan,China | human | blood | RDT+morphology+PCR |
| Po021 | *P.ovale* | Yunnan,China | human | blood | RDT+morphology+PCR |
| Po022 | *P.ovale* | Yunnan,China | human | blood | RDT+morphology+PCR |
| Po023 | *P.ovale* | Yunnan,China | human | blood | RDT+morphology+PCR |
| Po024 | *P.ovale* | Yunnan,China | human | blood | RDT+morphology+PCR |
| Po025 | *P.ovale* | Yunnan,China | human | blood | RDT+morphology+PCR |
| Po026 | *P.ovale* | Yunnan,China | human | blood | RDT+morphology+PCR |
| Po027 | *P.ovale* | Yunnan,China | human | blood | RDT+morphology+PCR |
| Po028 | *P.ovale* | Yunnan,China | human | blood | RDT+morphology+PCR |
| Po029 | *P.ovale* | Yunnan,China | human | blood | RDT+morphology+PCR |
| Po030 | *P.ovale* | Yunnan,China | human | blood | RDT+morphology+PCR |
| Po031 | *P.ovale* | Yunnan,China | human | blood | RDT+morphology+PCR |
| Po032 | *P.ovale* | Yunnan,China | human | blood | RDT+morphology+PCR |
| Po033 | *P.ovale* | Yunnan,China | human | blood | RDT+morphology+PCR |
| Po034 | *P.ovale* | Yunnan,China | human | blood | RDT+morphology+PCR |
| Po035 | *P.ovale* | Yunnan,China | human | blood | RDT+morphology+PCR |
| Po036 | *P.ovale* | Yunnan,China | human | blood | RDT+morphology+PCR |
| Co-inf001 | *P.malariae & P.ovale* | Yunnan,China | human | blood | RDT+morphology+PCR |
| Co-inf002 | *P.malariae & P.ovale* | Yunnan,China | human | blood | RDT+morphology+PCR |
| Co-inf003 | *P.malariae & P.ovale* | Yunnan,China | human | blood | RDT+morphology+PCR |
| Co-inf004 | *P.falciparum & P.ovale* | Yunnan,China | human | blood | RDT+morphology+PCR |
| Ld001 | *L. donovani* | Xingjiang,China | human | marrow | morphology+MLEE+PCR |
| Ld002 | *L. donovani* | Xingjiang,China | human | marrow | morphology+MLEE+PCR |
| Ld003 | *L. donovani* | Xingjiang,China | human | blood | RDT+morphology+PCR |
| Ld004 | *L. donovani* | Xingjiang,China | human | blood | RDT+morphology+PCR |
| Ld005 | *L. donovani* | Xingjiang,China | human | blood | RDT+morphology+PCR |
| Ld006 | *L. donovani* | Xingjiang,China | sandfly | whole | PCR |
| Ld007 | *L. donovani* | Xingjiang,China | sandfly | whole | PCR |
| Ld008 | *L. donovani* | Xingjiang,China | sandfly | whole | PCR |
| Ld009 | *L. donovani* | Xingjiang,China | sandfly | whole | PCR |
| Ld010 | *L. donovani* | Xingjiang,China | sandfly | whole | PCR |
| Ld011 | *L. donovani* | Xingjiang,China | dog | blood | PCR |
| Ld012 | *L. donovani* | Xingjiang,China | dog | blood | PCR |
| Ld013 | *L. donovani* | Sichuan,China | human | marrow | RDT+morphology+PCR |
| Ld014 | *L. donovani* | Sichuan,China | sandfly | whole | PCR |
| Ld015 | *L. donovani* | Sichuan,China | sandfly | whole | PCR |
| Ld016 | *L. donovani* | Sichuan,China | dog | blood | PCR |
| Ld017 | *L. donovani* | Sichuan,China | dog | blood | PCR |
| Li001 | *L. infantum* | Xingjiang,China | human | marrow | morphology+MLEE+PCR |
| Li002 | *L. infantum* | Xingjiang,China | human | blood | RDT+PCR |
| Li003 | *L. infantum* | Xingjiang,China | human | blood | RDT+PCR |
| Li004 | *L. infantum* | Xingjiang,China | human | blood | RDT+PCR |
| Li005 | *L. infantum* | Xingjiang,China | human | blood | RDT+PCR |
| Li006 | *L. infantum* | Xingjiang,China | human | blood | RDT+PCR |
| Li007 | *L. infantum* | Xingjiang,China | sandfly | whole | PCR |
| Li008 | *L. infantum* | Xingjiang,China | sandfly | whole | PCR |
| Li009 | *L. infantum* | Xingjiang,China | sandfly | whole | PCR |
| Li010 | *L. infantum* | Xingjiang,China | sandfly | whole | PCR |
| Li011 | *L. infantum* | Xingjiang,China | sandfly | whole | PCR |
| Li012 | *L. infantum* | Xingjiang,China | sandfly | whole | PCR |
| Li013 | *L. infantum* | Xingjiang,China | sandfly | whole | PCR |
| Li014 | *L. infantum* | Xingjiang,China | sandfly | whole | PCR |
| Li015 | *L. infantum* | Xingjiang,China | human | blood | RDT+PCR |
| Li016 | *L. infantum* | Xingjiang,China | human | blood | RDT+PCR |
| Li017 | *L. infantum* | Xingjiang,China | sandfly | whole | PCR |
| Li018 | *L. infantum* | Xingjiang,China | sandfly | whole | PCR |
| Li019 | *L. infantum* | Gansu,China | human | marrow | morphology+MLEE+PCR |
| Li020 | *L. infantum* | Gansu,China | dog | spleen | morphology+PCR |
| Li021 | *L. infantum* | Gansu,China | dog | blood | PCR |
| Li022 | *L. infantum* | Gansu,China | dog | blood | PCR |
| Li023 | *L. infantum* | Sichuan,China | human | marrow | morphology+PCR |
| Li024 | *L. infantum* | Sichuan,China | dog | blood | PCR |
| Li025 | *L. infantum* | Sichuan,China | dog | blood | PCR |
| Tg001 | *T.gondii* | United States | goat | DNA | morphology+PCR |
| Tg002 | *T.gondii* | France | human | DNA | morphology+PCR |
| Tg003 | *T.gondii* | Canada | Cougar | DNA | morphology+PCR |
| Tg004 | *T.gondii* | Brazil | cat | DNA | morphology+PCR |
| Tg005 | *T.gondii* | France | human | DNA | morphology+PCR |
| Tg006 | *T.gondii* | Qinghai,China | sheep | DNA | morphology+PCR |
| Tg007 | *T.gondii* | United States | sheep | DNA | morphology+PCR |
| Tg008 | *T.gondii* | United States | cat | DNA | morphology+PCR |
| Tg009 | *T.gondii* | United States | Deer | DNA | morphology+PCR |
| Tg010 | *T.gondii* | Costa Rica | Toucan | DNA | morphology+PCR |
| Tg011 | *T.gondii* | Yunnan,China | cat | blood | morphology+PCR |
| Tg012 | *T.gondii* | Yunnan,China | cat | blood | morphology+PCR |
| Tg013 | *T.gondii* | Yunnan,China | cat | blood | morphology+PCR |
| Tg014 | *T.gondii* | Yunnan,China | cat | blood | morphology+PCR |
| Tg015 | *T.gondii* | Yunnan,China | cat | blood | morphology+PCR |
| Tg016 | *T.gondii* | Guangdong,China | cat | blood | PCR |
| Tg017 | *T.gondii* | Guangdong,China | dog | blood | PCR |
| Tg018 | *T.gondii* | Guangdong,China | cat | blood | PCR |
