# Supplementary material for: DNA Microarray Detection of 18 Important Human Blood Protozoan Species
Source: PLoS Negl Trop Dis. 2016 Dec 2;10(12):e0005160. doi: 10.1371/journal.pntd.0005160 (PMC5135439; doi:10.1371/journal.pntd.0005160)
Supplement: S3 Table — (DOCX) [file pntd.0005160.s010.docx]

**Table S3.** Specific premiers for blood protozoa amplification

| **NO.** | **Blood protozoa** | **Name of specific premiers** | **Sequences of specific premiers** | **The length of gene fragment（bp)** | **Target gene** |
| --- | --- | --- | --- | --- | --- |
| 1 | *B.microti* | Bmi-F | TTCCAGCTCCAATAGCGT | 1090 | 18s |
|  |  | Bmi-R | AATTCACCGGATCACTCG |  |  |
| 2 | *B. divergens* | Bdi-F | CATTCAAGTTTCTGACCCAT | 1125 | 18s |
|  |  | Bdi-R | GCAGCCCAGGACATCTAA |  |  |
| 3 | *B. duncani* | Bdu-F | GAGAAACGGCTACCACAT | 1365 | 18s |
|  |  | Bdu-R | CAGGTTCACCTACGGAAA |  |  |
| 4 | *B. venatorum* | Bve-F | TGAGAAACGGCTACCACA | 1618 | 18s |
|  |  | Bve-R | GACGAGGCATAACCCAAT |  |  |
| 5 | *P. vivax* | Pvi-F | ACAAGGTTTCCGTAGGTG | 232 | 18s+its |
|  |  | Pvi-R | TTCATTTTACTGCCGATTA |  |  |
| 6 | *P. falciparum* | Pfa-F | ATTGACGGAAGGGCACCA | 555 | 18s+its |
|  |  | Pfa-R | CGGCGGAGGAAAAGTATG |  |  |
| 7 | *P. knowlesi* | Pkn-F | ATTTCTTACGCATACTAC | 1039 | coxI |
|  |  | Pkn-R | TATAACCTTACGGTCTGT |  |  |
| 8 | *P. malariae* | Pma-F | CCTTACAGACGCTTCCAG | 437 | coxI |
|  |  | Pma-R | GGCAGTTTGTTCCCTATC |  |  |
| 9 | *P.ovale* | Pov-F | TTCATCTCCAAGCCTCAT | 1205 | coxI |
|  |  | Pov-R | TGGCACCAATAGATAATACA |  |  |
| 10 | *L. donovani* | Ldo-F | TCCCTGCGAGGAAGAC | 1229 | 18s |
|  |  | Ldo-R | CAAGTTTGACGCCACATA |  |  |
| 11 | *L. gerbilli* / *L. tropica* | Lei-F | TGTGTACCTCTGCTCGTGTG | 756 | 18s |
|  |  | Lei-R | GTGACMACGTGAGTCGYGTGT |  |  |
| 12 | *L. gerbilli* | Lge-F | CTCTGTGCGACCATTGCG | 1681 | fh |
|  |  | Lge-R | TCCGTCACCTGCTTGCTG |  |  |
| 13 | *L. tropica* | Ltr-F | CACCGACCCCGCCTACCACT | 1447 | mspC |
|  |  | Ltr-R | GTCATTCCACCGCAGCAT |  |  |
| 14 | *L. infantum* | Lin-F | CATTCTATGCTTGGGGACC | 1421 | 18s |
|  |  | Lin-R | CCTGTAGCGATGCTGACG |  |  |
| 15 | *L. aethiopica* | Lae-F | CGAAACGCCAAGCTAATA | 1503 | 18s |
|  |  | Lae-R | CCAAACAAATCACTCCACC |  |  |
| 16 | *T. b. rhodesiense*/ *T. b. gambiense* | Try-F | TTCTACGGAGGGCAGCAGGC | 1111 | 18s |
|  |  | Try-R | CTGTAACCTCAAAGCTTTCGCGT |  |  |
| 17 | *T. b. rhodesiense* | Tbr-F | CTCTGCGCTTTTGTCTGA | 651 | p67 |
|  |  | Tbr-R | TACGCCCCACTGCTTTCC |  |  |
| 18 | *T. b. gambiense* | Tbg-F | TTTATCGGAAAACCCAACG | 1728 | hp |
|  |  | Tbg-R | CTACGCAGCCCAGAAACC |  |  |
| 19 | *T. cruzi* | Tcr-F | CGAACAACTGCCCTATCA | 1932 | 18s |
|  |  | Tcr-R | ACGACTTTTGCTTCCTCT |  |  |
| 20 | *T.gondii* | Tgo-F | GATAATTACAATACATGGTC | 1020 | coxI |
|  |  | Tgo-R | TAGAAGACAAATCCACAT |  |  |
