# Supplementary material for: DNA Microarray Detection of 18 Important Human Blood Protozoan Species
Source: PLoS Negl Trop Dis. 2016 Dec 2;10(12):e0005160. doi: 10.1371/journal.pntd.0005160 (PMC5135439; doi:10.1371/journal.pntd.0005160)
Supplement: S4 Table — (DOCX) [file pntd.0005160.s011.docx]

**Table S4.** Probes for blood protozoa detection

| **NO.** | **Name of genus or species** | **Sequences of probes** |
| --- | --- | --- |
| 1 | *Babesia* | CCCGCCCGTCGCTCTCTACCGATCGATGTGATCCGGTGAA |
| 2 | *Babesia* | AAGTTTTGTGAACCTTATCACTTAAAGGGAAGGAGAAGTC |
| 3 | *Babesia* | TGTGAACCTTATCACTTAAAGGGAAGGAGAAGTCGTAACA |
| 4 | *Babesia* | ataaacttgcgaatcgcatgcttttgcggcgatggcccattcaagtttctgacccatcag |
| 5 | *Babesia* | tcgatgccttttggcggcgtttattagcttttaaaccatcctcggtttcggtgattcata |
| 6 | *Babesia* | ccagtttttaccttgagaaaactagagtgtttcaaacaggcaatcgccttgaatactgc |
| 7 | *Babesia* | attcaacgagtttttccttggccgtcgggtccgggtaatcttacagtatgcatcgtgatg |
| 8 | *Babesia* | CAAAGTGCGATATCCAGCATGATTTGCAACTTCTTGCGAT |
| 9 | *Babesia* | TGATTTGCAACTTCTTGCGATTGCTAGACCTCTGAACGTA |
| 10 | *Babesia* | AACTCCCAGCGATGGATGCCTCGGCTCGCGCCTCGATGAA |
| 11 | *Babesia* | ACTCCCAGCGATGGATGCCTCGGCTCGCGCCTCGATGAAG |
| 12 | *B. microti* | cttgccattaatctcgcttccgagcgttttttattggcttggcatctttctggattttg |
| 13 | *B. microti* | tgccattaatctcgcttccgagcgttttttattggcttggcatctttctggattttgta |
| 14 | *B. divergens* | TGACTCATGTCGAGATTGCACTTCGCTTTTGCGTGGGATG |
| 15 | *B. divergens* | TGCGTGGTGTTAATATTGACTGATGTCGAGATTGCACTTC |
| 16 | *B. divergens* | GCGTGGTGTTAATATTGACTGATGTCGAGATTGCACTTCG |
| 17 | *B. divergens* | CGTGGTGTTAATATTGACTGATGTCGAGATTGCACTTCGC |
| 18 | *B. divergens* | GTGGTGTTAATATTGACTGATGTCGAGATTGCACTTCGCT |
| 19 | *B. venatorum* | CTGTGTGGGTTCCGCTTTGTGGATTCTCGCATTGTGGCCT |
| 20 | *B. venatorum* | CAGCTGCGCCAGTTTTGGTGCGGTTCACTGTGTGGGTTCC |
| 21 | *B. venatorum* | CAGTTTTGGTGCGGTTCACTGTGTGGGTTCCGCTTTGTGG |
| 22 | *B. venatorum* | TGGGTTCCGCTTTGTGGATTCTCGCATTGTGGCCTCGTGC |
| 23 | *B. duncani* | GTTCGCCTGGTGGCTTACCTCTGGCGGTGGTTCTCCATTT |
| 24 | *Plasmodium* | AATATTGGAGCACGTAAGAAAGTGAAATCGGCTAGCTGTG |
| 25 | *Plasmodium* | TTTCTTAATATTCTTATTGGCTTTCGGGATCGGAGTTACG |
| 26 | *Plasmodium* | ATATTCTTATTGGCTTTCGGGATCGGAGTTACGATTAATA |
| 27 | *Plasmodium* | TTTGGCTTTCGGGATCGGAGTTACGATTAATAGGAGTAGC |
| 28 | *Plasmodium* | ACACGGGAAAACTCACTAGTTTAAGACAAGAGTAGGATTG |
| 29 | *Plasmodium* | TCTTTTCTTGATTTCTTGGATGGTGATGCATGGCCCGTTT |
| 30 | *Plasmodium* | ATTAGTAGAACAGGGAAAAGGATATTTTAATAAAAATATC |
| 31 | *Plasmodium* | AATAAAAATATCCTAATTTGATTACTGAATAAATGTATAGT |
| 32 | *Plasmodium* | CAAGTCTACGACTGAGCAATATCTCTGTACACTGATTAGA |
| 33 | *Plasmodium* | TACTGTTCGGAGGAACTACAGGTGTTAGCCTAGGTAATGC |
| 34 | *P. vivax* | gcgcaggaaatcccaacaattttcaccgatcaatcttttgcataaatgtgtcatgtctgc |
| 35 | *P. vivax* | ggtgcagcggcgaattgtgatttaattattgtttgttgaggtgtttaacgatgcttcgat |
| 36 | *P. falciparum* | ATTAGTAGAACAGGGAAAAGGATATTTTAATAAAAATATCCTAATTTGATTACTGAATAA |
| 37 | *P. falciparum* | AATAAAAATATCCTAATTTGATTACTGAATAAATGTATAGTTACCTATGTTCAATTTCAA |
| 38 | *P. knowlesi* | ACAAGTCTACGATAAGACAATATCTCTGTACACTGATTAG |
| 39 | *P. knowlesi* | CAAGTCTACGATAAGACAATATCTCTGTACACTGATTAGA |
| 40 | *P. knowlesi* | AGTCTACGATAAGACAATATCTCTGTACACTGATTAGAAC |
| 41 | *P. knowlesi* | GTCTACGATAAGACAATATCTCTGTACACTGATTAGAACA |
| 42 | *P. malariae* | AAGAGTGGAAAATAATGTCCAGCCAACACCATCCAATTTG |
| 43 | *P. malariae* | GAGTGGAAAATAATGTCCAGCCAACACCATCCAATTTGAT |
| 44 | *P.ovale* | CATATGTATACCACAGGTTTAGAGATAGACACTAGAGCTT |
| 45 | *P.ovale* | TACTGTTCGGAGGAACTACAGGTGTTATTTTAGGTAATGC |
| 46 | *Leishmania* | GAAGGGTTGCTGTGTGCGTGCCATTCCGTGAAGCGGCAT |
|  |  | ATCTTTTCTATTCGGCCTTTACCGGCCACCCACGGGAATATCCTCAGCACGTTTTCTGTT |
|  |  | TTTAAAGGTCTATTGGAGATTATGGAGCTGTGCGACAAGTGCTTTCCCATCGCAACCTCG |
| 47 | *Leishmania* | TCCTACGTCGATCTCTTTCAGTCGCACGGCGCGCAGTAC |
|  |  | TTTACCACCTTACGTATCTTTTCTATTCGGCCTTTACCGGCCACCCACGGGAATATCCTC |
|  |  | CTCGTTTAAAGGTCTATTGGAGATTATGGAGCTGTGCGACAAGTGCTTTCCCATCGCAAC |
| 48 | *Leishmania* | GAGCGTGTGTGGATAACGTTTTGATGCGGGGCTCACATTG |
| 49 | *Leishmania* | CGTGTGTGGATAACGTTTTGATGCGGGGCTCACATTGAGA |
| 50 | *Leishmania* | GCCAAGAGGAGGCGTGTGTTTGTGCCACCACCACGTACTT |
| 51 | *Leishmania* | GCGCGTGTGTGGATAACGTTTTGATGCGGGGCTCACATTG |
| 52 | *Leishmania* | TGGAAGCCAAGAGGAGGCGTGTGTTTGTGCCACCACCACG |
| 53 | *Leishmania* | GCCAAGAGGAGGCGTGTGTTTGTGCCACCACCACGTACTC |
| 54 | *L. gerbilli* | CGACAAACAAGGCGTACCTGTACCAGGAAACCAAGGCGTT |
| 55 | *L. gerbilli* | TTGCGGTGTCTTGCAGCGCTGATCGCCAGATTCTTGCGCA |
| 56 | *L. gerbilli* | GTGTCTTGCAGCGCTGATCGCCAGATTCTTGCGCACATCA |
| 57 | *L. gerbilli* | TCCTACGTCGATCTCTTTCAGTCGCACGGCGGCAGCTACA |
| 58 | *L. gerbilli* | CATCTACATTGAGCAGCTTGAGCAGAACCCGGCGCAGTAC |
| 59 | *L. gerbilli* | TACCAGGAAACCAAGGCGTTGCTGAACCCCAAATCGCTGC |
| 60 | *L. donovani* | GAAGGGTTGCTGTGTGCGTGCCATTCCGTGAAGCGTCATT |
| 61 | *L. donovani* | CTGTGTGCGTGCCATTCCGTGAAGCGTCATTCCTATGTCC |
| 62 | *L. donovani* | GAAGCGTCATTCCTATGTCCGACACTGAAATGCGTCCGCG |
| 63 | *L. donovani* | ATGCGTCCGCGATTGTGTGGATTGTGTGGAAGTGTCTGGG |
| 64 | *L. donovani* | GTCCGCGATTGTGTGGATTGTGTGGAAGTGTTTGGGCGAC |
| 65 | *L. donovani* | CCTACGCAAAGGTACAAAAGCAAGCGCATCGAGCCAGACG |
| 66 | *L. donovani* | AAGACCCTCGCGGGAGCATTGCTTCGCGTCCCAGTACTGA |
| 67 | *L. donovani* | GAGGAAGACCCTCGCGGGAGCATTGCTTCGCGTCCCAGTA |
| 68 | *L. donovani* | CTCGCGGGAGCATTGCTTCGCGTCCCAGTACTGAGCATCC |
| 69 | *L. donovani* | TCGCGGGAGCATTGCTTCGCGTCCCAGTACTGAGCATTCC |
| 70 | *L. infantum* | ACTAGAGGCCGGTCCATCTTAGGCTCACGCCATAAGCTTC |
| 71 | *L. infantum* | GCAATCACTAATTGGGCTTAAGCGTCATCCATTTGCGGGG |
| 72 | *L. infantum* | CGGCTCACATAGCCACCGTCCAACTGTCCATATCCACCAA |
| 73 | *L. infantum* | AGCCACCGTCCAACTGTCCATATCCACCAACACCCTTTTC |
| 74 | *L. tropica* | GCTCCATTCATGGACTACTGCCCGGTCGTTATCGGCTACG |
| 75 | *L. tropica* | GCCATTCATGGACTACTGCCCGGTCGTTATCGGCTACGCT |
| 76 | *L. tropica* | CCATTCATGGACTACTGCCCGGTCGTTATCGGCTACGCTT |
| 77 | *L. aethiopica* | AGGTTTGTTCCTGGTCGTCCCGTCCATGTCGGATTTGGTG |
| 78 | *L. aethiopica* | TTTGGTGACCCAGGCCCTTGCAGCCCGTGAACATTCAAAG |
| 79 | *L. aethiopica* | GGGCAACCATCGTCGTGAGACGCCCAGCGAATGAATGACA |
| 80 | *L. aethiopica* | CCGTTACGCCTTTTCAACTCACGGCCTCTAGGAATGAAGG |
| 81 | *L. aethiopica* | GTTTACCCTGTGTCAGCACCGCGCCCGCTTTTACCAACTT |
| 82 | *L. aethiopica* | CCTGTGTCAGCACCGCGCCCGCTTTTACCAACTTACGTAT |
| 83 | *Trypanosoma* | TGCATGGGTTGATACCCCGCTTTTGGTCAAGGTGGAGTGA |
| 84 | *Trypanosoma* | GACAAGCGGCTGGGTGGTTATTCCACACACACACACACAC |
| 85 | *Trypanosoma* | GTGTGGCACTCGTCGCCTTTGTGGGAAATCCGTGTGGCAC |
| 86 | *Trypanosoma* | GCCTTTGTGGGAAATCCGTGTGGCACGTGTTTTGTGTGTT |
| 87 | *Trypanosoma* | AAATCCGTGTGGCACGTGTTTTGTGTGTTGTTGGCAGAGA |
| 88 | *Trypanosoma* | GCACGTGTTTTGTGTGTTGTTGGCAGAGACTTCGGTCTTT |
| 89 | *Trypanosoma* | TGTGTTGTTGGCAGAGACTTCGGTCTTTTGCCCTTCGCAT |
| 90 | *Trypanosoma* | AACCAAAGTGTGGGGATCGAAGACTGATTAGAGACCATTG |
| 91 | *Trypanosoma* | TCCCTGGCTTGTTGTTCCTCGTCTCGCCAATTGAATATAT |
| 92 | *Trypanosoma* | TCTTTCCCACATGTGTCATGCCTTCCCTCAACTAGCTGTTA |
|  |  | GATTTTTTCCCCAACGGTGGTCGTCATCCTTCTTTTTACAGGCCCCTTCTCTGCGGGATT |
|  |  | TAGACCCACTTGGAACCGAGTATTGCAATTATTGGTCGCGCAACGAGGAATGTCTCGTAG |
|  |  | TAGACCCACTTGGGACCGAGTATTGCAATTATTGGTCGCGCAACGAGGAATGTCTCGTAA |
| 93 | *Trypanosoma* | GGCGCAATGGTTCAGTCCCATCCACTGCGGACTTGGTAG |
|  |  | ATGATTAGAGACCATTGTAGTCCACACTGCAAACGATGACACCCATGAGTTGGGAAGTTT |
|  |  | GCATGTCATGCATGCCAGAGGGCGCCCGTGATTCTTTACTGTGACTAAAAAAGTGTGACC |
|  |  | CAGAAACAAAAAACACGGGAGTGGTACCTCTCTGATTTTCGCATGTCATGCATGCCAGGG |
| 94 | *Trypanosoma* | TCTTTCCCACATGTGTCATGCCTTCCCTGGACTTGGTAGA |
|  |  | GATGATTAGAGACCATTGTAGTCCACACTGCAAACGATGACACCCATGAATTGGGAAGTT |
|  |  | CTGTGACTAAAAAAGTGCGACCAAAGCAGTCCGCCGACTTGAATTACAAAGCATGGGATA |
|  |  | TACTGTGACTAAAAAAGTGCGACCAAAGCAGTCCGCCGACTTGAATTACAAAGCATGGGA |
| 95 | *T. cruzi* | TCTTTCCCACATGTGTCATGCCTTCCCTCAACTCAGTATG |
| 96 | *T. cruzi* | GGCGCAATGGTTTAGTCCCATCCACTTCGGACTTGGTAGA |
| 97 | *T. cruzi* | TCATCCCGTTCCTCGTCTCGCCAATGAATATATTAAATTT |
| 98 | *T. b. rhodesiense* | GCCCCTGCTGACGAACGGTTGGATCGGATGAAGCTGTTAA |
| 99 | *T. b. rhodesiense* | ACTCGTGCCCCTGCTGACGAACGGTTGGATCGGATGAAGC |
| 100 | *T. b. rhodesiense* | TGCCCCTGCTGACGAACGGTTGGATCGGATGAAGCTGTTA |
| 101 | *T. b. gambiense* | GCGAGTGCCTTCGCAATTCTGTTCCGTTACTTAGCGCTGC |
| 102 | *T. b. gambiense* | ACACGAGACCTCCGAGCATCTTTCTTGTAAACCCACGGGC |
| 103 | *T. b. gambiense* | GCAGGTGGCATGGAAGTTCTGAAGGGCCTAAAGCGTACAG |
| 104 | *T.gondii* | GTTCTTAATGCCGGCTTTGTACGGAGGATATGGTAACTTC |
| 105 | *T.gondii* | TGCCGGCTTTGTACGGAGGATATGGTAACTTCTTTGTACC |
| 106 | *T.gondii* | GTGGTTCGGAAGTCGTTTTCCCAAGAACTAACGCGATCTC |
| 107 | *T.gondii* | TGGTGGTTCGGAAGTCGTTTTCCCAAGAACTAACGCGATC |
